# Computational Predictive and Electrochemical Detection of Metabolites (CP-EDM) of Piperine

**DOI:** 10.3390/molecules29102406

**Published:** 2024-05-20

**Authors:** Ridho Asra, Ana P. R. Povinelli, Gabriel Zazeri, Alan M. Jones

**Affiliations:** 1 School of Pharmacy, College of Medical and Dental Sciences, University of Birmingham, Birmingham B15 2TT, UK; rxa240@student.bham.ac.uk; 2 Departament of Physics, Instituto de Biociências, Letras e Ciências Exatas (IBILCE), UNESP, Rua Cristovão Colombo 2265, São José do Rio Preto 15054-000, SP, Brazil; 3 Departament of Physics, Universidade Federal de Roraima (UFRR), Av. Cap. Ene Garcês, 2413—Aeroporto, Boa Vista 69310-000, RR, Brazil

**Keywords:** piperine, metabolite, electrochemical, detection, predictive, CP-EDM

## Abstract

In this article, we introduce a proof-of-concept strategy, Computational Predictive and Electrochemical Detection of Metabolites (CP-EDM), to expedite the discovery of drug metabolites. The use of a bioactive natural product, piperine, that has a well-curated metabolite profile but an unpredictable computational metabolism (Biotransformer v3.0) was selected. We developed an electrochemical reaction to oxidize piperine into a range of metabolites, which were detected by LC-MS. A series of chemically plausible metabolites were predicted based on ion fragmentation patterns. These metabolites were docked into the active site of CYP3A4 using Autodock4.2. From the clustered low-energy profile of piperine in the active site, it can be inferred that the most likely metabolic position of piperine (based on intermolecular distances to the Fe-oxo active site) is the benzo[*d*][1,3]dioxole motif. The metabolic profile was confirmed by comparison with the literature, and the electrochemical reaction delivered plausible metabolites, vide infra, thus, demonstrating the power of the hyphenated technique of tandem electrochemical detection and computational evaluation of binding poses. Taken together, we outline a novel approach where diverse data sources are combined to predict and confirm a metabolic outcome for a bioactive structure.

## 1. Introduction

An understanding of the major circulating metabolites that are generated from a parent bioactive molecule is of critical importance in drug discovery campaigns [1]. One powerful and emerging approach to detect and identify drug metabolites is the use of electrochemical techniques [2,3,4,5,6,7,8,9,10,11,12,13,14]. An alternative approach is to computationally predict via docking how a drug might interact with a cytochrome P_450_ enzyme [15,16,17]. However, approaches that interlink these two complementary techniques are not fully delineated at present.

We have a long-standing interest in the chemistry [18] and molecular interactions [19] of the bioactive natural product, piperine. Piperine (molecular weight of 285 g/mol) is an alkaloid found in various plant species, including the Piperacea family (*Piper tuberculatum*) in the Brazilian Amazon region [20]. Piperine exhibits significant pharmacological effects such as anti-inflammatory, anti-carcinogenic, anti-microbial, and anti-parasitic properties. Furthermore, piperine plays a role in enhancing the bioavailability of numerous drugs by inhibiting drug-metabolizing enzymes, thereby delaying the clearance of these drugs [21].

In vivo studies in rats [22] revealed that after oral administration, piperine reached its highest concentration (C_max_) in serum and various organs within 6 h. Piprerine’s presence remained detectable for up to 96 h post-administration, being metabolized before excretion from the organism. Piperine is a potent inhibitor of cytochrome CYP3A4 and related familial isoforms [23,24], but to the best of our knowledge, the full biological mechanism by which piperine is metabolized remains unknown [25]. There is, however, a body of evidence of typical piperine metabolites to enable confirmation by comparison vida infra [26,27,28,29].

The CP-EDM study proposed is a hyphenated technique for computational prediction and electrosynthesis to expedite the metabolic reactivity profile of bioactive molecules. Electrosynthesis is a green methodology that enables the synthesis and identification of drug metabolites based on their oxidation analytical profile (cyclic voltammetry analytical measurements) [4]. Related studies in chlorpromazine demonstrated that electrosynthesis approaches can mimic human metabolism by generating the same metabolites from in vitro/in vivo and clinical studies [30]. Understanding how drugs interact with the metabolizing enzyme is the key to predicting their metabolism in humans. This can be predicted by using computational platforms that can simulate these interactions to predict which enzymes are likely to metabolize a particular drug, how the drug might interact with heme Fe in the cytochrome enzymes, and which regions of the drug are most likely to undergo metabolism. The integration of electrosynthesis was employed to reveal how a bioactive structure metabolizes in the human body. This would could minimize time-consuming biological studies of unsuitable compounds, thus reducing the cost of one of the key steps in drug development. In this study, electroanalysis via a cyclic voltammetry (CV) study was employed to understand the redox behavior of piperine. The information from the CV study was used to optimize and conduct the electrosynthesis reaction, and the metabolites generated are analyzed by using LC-MS alongside a computational study to identify the potential oxidation site of piperine for understanding and predicting the drug metabolite resulting from the reaction.

CP-EDM studies offer a technique in metabolite prediction for drug development and hold promise in advancing toxicology research in drug development. By comparison with the in vitro and in vivo studies, the results may find use in predicting and identifying potential drug metabolites. This study aids in assessing the safety and effectiveness of the drug via its metabolite profile, which is vital for regulatory approval and clinical use.

## 2. Results and Discussion

### 2.1. Electrochemical Behavior of Piperine: Cyclic Voltammetry Studies

Prior to the electrochemical reaction, the CV behavior of piperine was measured [31]. We previously showed that the voltammetric behavior of a drug molecule is inversely correlated to its in situ metabolic half-life [32]. Using ferrocene (Fc/Fc^+^) as an internal standard for the *pseudo* Ag/AgCl wire reference electrode (RE), a glassy carbon electrode (GCE) as the working electrode (WE), and a platinum wire as the counter electrode (CE), the voltammetric behavior of piperine was analysed.

**Figure 1:**
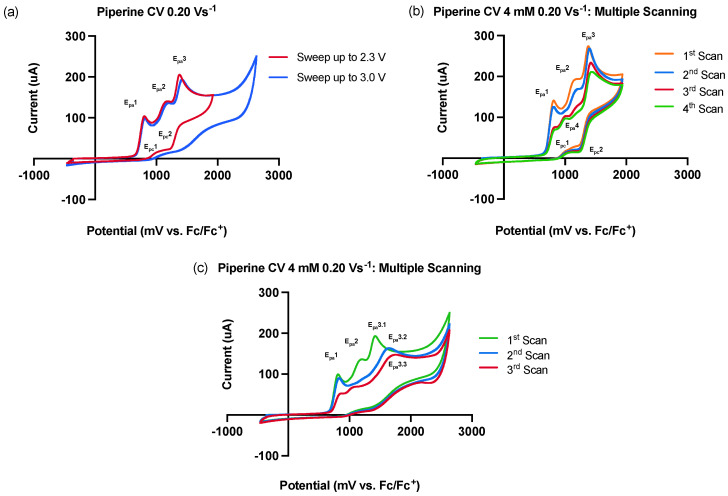
Cyclic voltammetry behavior of piperine, GCE (WE), Pt (CE), Ag/AgCl (*pseudo* RE) referenced to Fc/Fc^+^. Electrolyte: TBAPF_6_; solvent: MeCN. (**a**) Piperine CV behaviors in two different potential scannings—Conditions 1: start potential = 0 V_reff_; upper vertex potential = 2.30 V_reff_; lower vertex potential = −0.10 V_reff_; stop potential = 0 V_reff_; number of scans = 1; ʋ: 0.20 Vs^−1^; Step: 0.00244 V. [E_pa_1 (E_p_ = 808 mV; i_p_ = 104 µA); E_pa_2 (E_p_ = 1174 mV; i_p_ = 141 µA); E_pa_3 (E_p_ = 1386 mV; i_p_ = 205 µA); E_pc_1 (E_p_ = 866 mV; i_p_ = 1.95 µA); E_pc_2 (E_p_ = 1223 mV; i_p_ = 24 µA)]. Conditions 2: start potential = 0 V_reff_; upper vertex potential = 3.00 V_reff_; lower vertex potential = −0.10 V_reff_; stop potential = 0 V_reff_; number of scans = 1; ʋ: 0.20 Vs^−1^; Step: 0.00244 V. [E_pa_1 (E_p_ = 818 mV; i_p_ = 99 µA); E_pa_2 (E_p_ = 1191 mV; i_p_ = 136 µA); E_pa_3 (E_p_ = 1425 mV; i_p_ = 194 µA); E_pc_1 (E_p_ = 930 mV; i_p_ = −0.85 µA); E_pc_2 (E_p_ = 1379 mV; i_p_ = 20 µA)]. (**b**) Effects of multiple scanning up to +2.3 V on piperine cyclic voltammetry behavior—Conditions: start potential = 0 V_reff_; upper vertex potential = 2.3 V_reff_; lower vertex potential = −0.10 V_reff_; stop potential = 0 V_reff_; number of scans = 4; ʋ: 0.20 Vs^−1^; Step: 0.00244 V. [E_pa_1 (E_p_ = 820 mV; i_p_ = 141 µA); E_pa_2 (E_p_ = 1168 mV; i_p_ = 193 µA); E_pa_3 (E_p_ = 1383 mV; i_p_ = 274 µA); E_pc_1 (E_p_ = 891 mV; i_p_ = 0.84 µA); E_pc_2 (E_p_ = 1213 mV; i_p_ = 30 µA); E_pa_4 (E_p_ = 1013 mV; i_p_ = 104 µA)]. (**c**) Effects of multiple scanning up to 3.0 V on piperine cyclic voltammetry behavior—Conditions: start potential = 0 V_reff_; upper vertex potential = 3.0 V_reff_; lower vertex potential = −0.10 V_reff_; stop potential = 0 V_reff_; number of scans = 3; ʋ: 0.20 Vs^−1^; Step: 0.00244 V. [E_pa_1 (E_p_ = 818 mV; i_p_ = 99 µA); E_pa_2 (E_p_ = 1203 mV; i_p_ = 136 µA); E_pa_3.1 (E_p_ = 1427 mV; i_p_ = 193 µA); E_pa_3.2 (E_p_ = 1657 mV; i_p_ = 163 µA); E_pa_3.3 (E_p_ = 1739 mV; i_p_ = 148 µA)].

The CV profiles of piperine are shown in Figure 1. The electroanalysis procedure followed the general procedure (see Section 3) with configuration fixed with a 0.00244 V step potential and a start and stop potential of 0.0 V. From inspection of Figure 1a,b, piperine has three oxidation events (Epa1, Epa2, and Epa3) that occur at 808 mV, 1174 mV, and 1386 mV, respectively, and two reduction events (Epc1 and Epc2) at 877 mV and 1223 mV vs. Fc/Fc^+^. The existence of the oxidation and reduction events may correspond to the redox species present in the piperine structure in biological settings.

Changing to multiple scanning of potential waveforms provides several pieces of information, including the reversibility or irreversibility of an electrochemical reaction. To confirm the type of the piperine oxidation process, two different multiple scanning analyses were employed, as shown in Figure 1b,c. These studies reveal the multiple features of piperine CVs when using different applied potentials, and the reversibility of the oxidation wave depends on the reversibility of later oxidation waves. Specifically, if the oxidation process generates an unstable species that degrades, all subsequent processes will be affected. For example, when scanned with a positive applied potential, piperine has three oxidation events with two return reduction waves observed. For the next scanning, the Epa4 occurs at 1013 mV, which details the process that generates a new chemical species that does not appear in the first scanning and increases for the subsequent scanning, as shown in Figure 1b. However, if the product after oxidation in Epa3 is unstable in solution, there will not be any product remaining during the return scan. In other words, the resulting oxidation product from Epa3 undergoes an irreversible oxidation process or decomposition. The irreversibility of the oxidation process appears in the multiple scanning analysis up to +3.0 V, as shown in Figure 1c. The Epa3 shift to a more positive potential for the subsequent scanning from 1427 mV to 1657 mV to 1739 mV. This means new chemical intermediate species are present after the first scann, leading to the different CV profiles of Epa3 shown in the second and third scans. The result suggests the irreversibility of the electrochemical properties system being studied for piperine.

**Figure 2:**
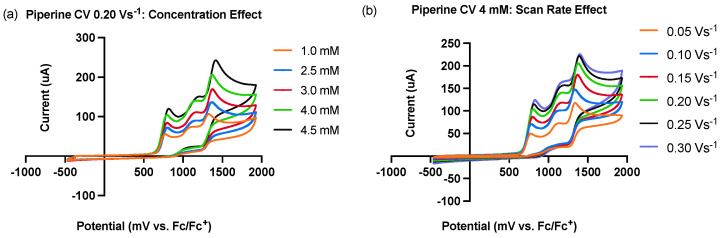
Cyclic voltammetry behavior of piperine (scan rate and concentrations varied against E_p_1). (**a**) Effects of piperine concentration on cyclic voltammetry behavior—Conditions: start potential = 0 V_reff_; upper vertex potential = 2.3 V_reff_; lower vertex potential = −0.10 V_reff_; stop potential = 0 V_reff_; number of scans = 1; ʋ: 0.20 Vs^−1^; Step: 0.00244 V. [1.0 mM (E_p_ = 776 mV; i_p_ = 56 µA); 2.5 mM (E_p_ = 801 mV; i_p_ = 72 µA); 3.0 mM (E_p_ = 805 mV; i_p_ = 84 µA); 4.0 mM (E_p_ = 798 mV; i_p_ = 104 µA); 4.5 mM (E_p_ = 825 mV; i_p_ = 120 µA)]. (**b**) Effects of piperine scan rate on cyclic voltammetry behavior—Conditions: start potential = 0 V_reff_; upper vertex potential = 2.3 V_reff_; lower vertex potential = −0.10 V_reff_; stop potential = 0 V_reff_; number of scans = 1; ʋ: 0.05–0.30 Vs^−1^; Step: 0.00244 V. [0.05 Vs^−1^ (E_p_ = 783 mV; i_p_ = 49 µA); 0.10 Vs^−1^ (E_p_ = 791 mV; i_p_ = 71 µA); 0.15 Vs^−1^ (E_p_ = 811 mV; i_p_ = 86 µA); 0.20 Vs^−1^ (E_p_ = 812 mV; i_p_ = 104 µA); 0.25 Vs^−1^ (E_p_ = 825 mV; i_p_ = 114 µA); 0.30 Vs^−1^ (E_p_ = 832 mV; i_p_ = 124 µA)].

Figure 2 depicts the concentration and scan rate effect of piperine CV studies. Figure 2a shows that the peak current (ip) of piperine increases with increasing analyte concentration because there are more redox-active species available to undergo oxidation at the electrode surface. With increasing scan rate (Figure 2b), the electrode potential changes more rapidly, and the rate of electron transfer reactions increases, leading to a larger peak current. These results are consistent with the Randles–Sevcik equation and indicate the diffusion-controlled redox process. In this Randles–Sevcik equation at 25 °C [33], (ip = k n^3/2^ A $\sqrt{Dv}$ C), ip is the peak current, k is a constant of 2.69 × 10^5^ C/mol√v, n is the number of electrons, A is the electrode area (cm^2^), D is the analytes diffusion coefficient (cm^2^s^−1^), ν is the rate at which the potential is swept (Vs^−1^), and C is concentration.

To determine whether the electrochemical analysis is controlled by adsorption or diffusion, a scan rate study was performed using a scan rate from 0.05 to 0.30 Vs^−1^ with 0.05 Vs^−1^ as the increment constant, as shown in Figure 2b and Table 1. The peak potential positively shifts to a higher potential from 783 to 832 mV. The diffusion coefficient increased with an increase in the scan rate, which means higher scan rates are suitable for detecting redox species [34]. A higher diffusion coefficient was achieved at 0.2 Vs^−1^, which means this sweep rate is more effective in identifying a larger number of redox species because it will enhance the mass transport of the electroactive species to and from the electrode surface.

According to Table 2, the piperine electrochemical process is dominantly controlled by diffusion rather than an adsorption-controlled mechanism. This can be seen from the square root of the scan rate v^1/2^ (V/s)^1/2^ vs. peak current (Ip_1_) (µA) with the regression equation of a 0.9975 that has a higher proportional linearity relationship than scan rate (mVs^−1^) vs. peak current (Ip_1_) with the regression equation of 0.9792. Moreover, the diffusion-controlled mechanism can be identified from the slope of the Log of the scan rate (mVs^−1^) vs. the Log of the peak potential (Ep_1_) mV (slope: 0.03439), which is closer to 0.5 than 1. The electrochemical reaction of piperine is controlled by the mass transfer as the slope of the Log of the scan rate (mVs^−1^) vs. the Log of the peak current (Ip_1_) (µA) is close to 0.5 (slope: 0.5230).

The peak potential (Ep) depends on the scan rate or concentration if the oxidation peak potential shifts to more positive potentials, and it is non-scan rate dependent if the Ep remains relatively constant as the scan rate and concentration increase. To analyze these relationships, the scan rate (mVs^−1^) vs. the peak potential (Ep_1_) mV and the concentration vs. the peak potential (Ep_1_) mV were plotted. The processes are dependent on the scan rate and non-dependent on the concentration with the regression equation of 0.9983 (strong correlation) and 0.7492 (weak correlation), respectively.

### 2.2. A Comparison of Electrosynthesis and Hepatocyte Incubation Metabolites

**Scheme 1:**
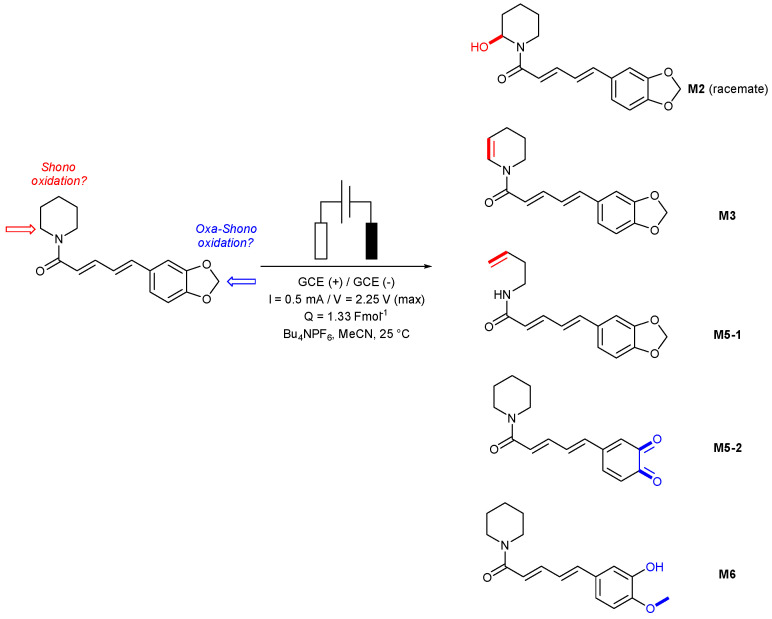
Electrochemical oxidation of piperine and LC−MS-identifiable metabolites. Key: Red indicates Shono-type oxidation reactions, and blue indicates Oxa-Shono-type oxidation reactions.

**Figure 3:**
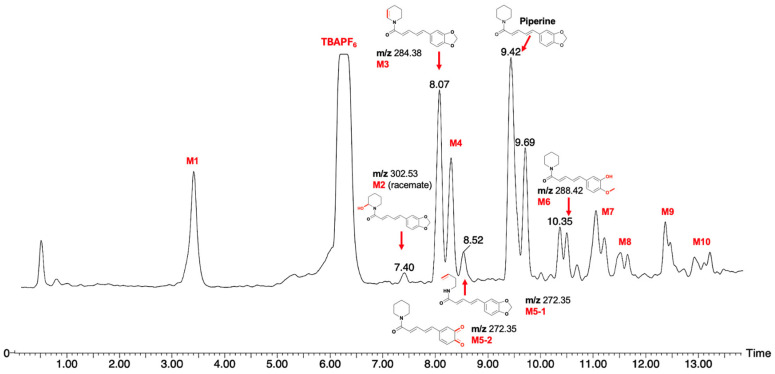
LCMS trace for the electrochemical reaction of piperine to a series of metabolites 1–10.

After establishing that piperine is both electrochemically active and has multiple oxidation events, we then employed our established protocols for Shono-type [35,36] and Oxa-Shono-type oxidations [37]. Piperine was reacted under electrochemical conditions, and the resulting series of products were analyzed by LC-MS (Scheme 1 and Figure 3).

Using the MS fragmentation patterns derived from the LCMS analysis (Figure 3), and via comparison with related electrochemical [26] and hepatocyte incubation data [27], the tentative structures of metabolites 2, 3, 5-1, 5-2, and 6 were determined, as shown in Scheme 1.

It should be noted that piperine exists as a *Z*,*Z*-conjugated alkene isomer, but upon electrochemical reaction, *E*,*Z* and/or *Z*,*E*-isomers form, as detected by thin-layer chromatography (TLC) and LC-MS analysis (see the Supplementary Materials) from a radical isomerism reaction in situ. A summary of the overlap between methods (electrochemical and hepatocyte incubation of piperine) and novel metabolites with this work is shown in Table 3.

### 2.3. Molecular Docking Approaches in Understanding Piperine Metabolism

Furthermore, other unassigned oxidative metabolites were produced in this reaction (see the Supplementary Materials). We next considered whether the confirmed or tentatively assigned oxidation metabolites would be possible from a predictive model. To enable this, we employed cytochrome (CYP3A4), a pivotal protein involved in the metabolism of numerous drugs, while piperine acts as an inhibitor of this protein [24]. CYP3A4 inhibition may lead to drug–drug interactions, toxicity, and other adverse effects, but it can also be beneficial and enhance the therapeutic efficiency of co-administered pharmaceuticals that are metabolized by CYP3A4.

**Figure 4:**
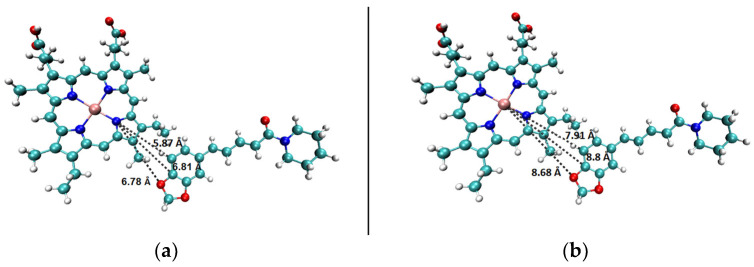
Docking of piperine into the active site of CYP3A4, resulting in the close proximity of piperine to (**a**) the closest heterocycle within porphyrin and (**b**) the closest interaction to the Fe atom. Both depictions indicate the benzo[*d*][1,3]dioxole motif of piperine as a predicted site for phase I oxidative metabolism in the body.

**Figure 5:**
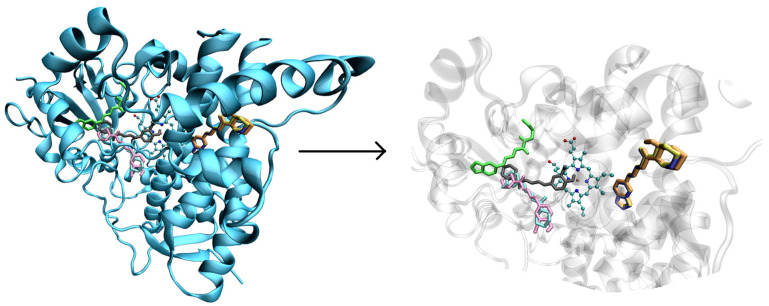
**Left**—General view of docked molecules into CYP3A4 (piperine in blue, M2 in orange, M3 in yellow, M5-1 in green, M5-2 in cyan, and M6 in black). **Right**—Zoomed-in region. The protein is a new cartoon outfit; molecules in licorice and the heme group in CPK.

The affinity energy function obtained from our docking of piperine (−8.73 kcal/mol) is very close to that reported in the literature (−8.55 kcal/mol), which gives added confidence in the predicted pose of piperine within the active pocket of CYP3A4 (Figure 4 and Figure 5) [37].

The *inter*molecular distances between the clusteredlow-energy binding pose of piperine (see the Supplementary Materials) and the *N* and *Fe* atoms of the heme Fe group in CYP3A4 are reported in Å in Figure 4. This model is indicative that the benzo[*d*][1,3]dioxole motif is more likely to engage with the heme Fe species and undergo an oxidation event in the body, for instance, metabolites M8, M12, M18, and M3, as detected in a hepatocyte incubation [27], which evaluates both phase I (oxidative) and phase II (conjugation) events. M5-2 and M6 from *our work* demonstrate an oxidative event in this fragment of piperine.

We next considered whether the metabolites identified in our electrochemical screen (Scheme 1) would feasibly exist in the CYP active pocket, post-metabolic oxidation. To achieve this, we explored the clustered binding patterns of our piperine metabolites compared to piperine itself within the cytochrome (Figure 5). The findings from molecular docking reveal that metabolites M2 and M3 dock precisely in the same region as piperine with an increase in the energy score for M3 and a decrease in the energy score for M2 (as shown in Table S1). This suggests that upon metabolism, the M2 metabolite exhibits diminished affinity for the protein.

**Figure 6:**
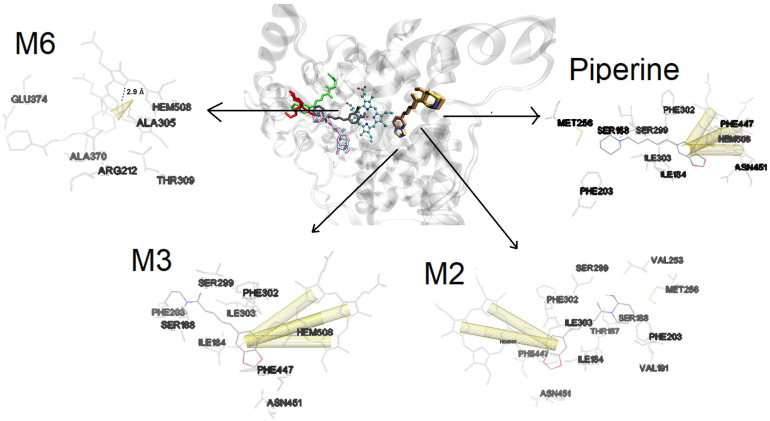
Visualization of the four docking poses (piperine, M2, M3, and M6) highlighting the π-π, cation–π (yellow regions), and hydrogen bond interactions (dashed bonds).

One of the highly specific interactions observed is π-π followed by the π–cation interaction, owing to its high level of molecular organization. These interactions are recognized as highly specific molecular recognition interactions in protein–ligand complexes, often considered complex identity interactions [38,39,40,41]. Analyzing the results of molecular docking (Figure 6), both metabolites M2 and M3 exhibit π-π interactions with the heme group of the protein. M3 presented an increase in binding affinity compared with piperine, while M2 presented a reduced affinity for the protein compared with piperine. These results strongly suggest that these piperine derivatives might act as protein inhibitors post-metabolism unless the decreased affinity caused M2 to vacate the binding site because of competition with other biological molecules. Moreover, besides the π-π interaction, the amino acids performed non-specific interactions with piperine, M2, and M3 with no hydrogen bonds presented.

Despite M6 not binding directly at the piperine site, it still engages in a π–cation interaction with the iron of the heme group and forms a hydrogen bond between the hydroxyl group of M6 and the heme group (2.9 Å). While M6 binds to the heme group, it is noteworthy that the π–cation interaction is less specific than π-π interactions, and the metabolite does not bind to the piperine interaction site. This suggests that this metabolite may not have the potential to inhibit the protein. Metabolites M5-1 and M5-2 do not interact with the heme group in the two lowest energy poses. The only specific interaction observed is a hydrogen bond formed by metabolite M5-2 with R212 (2.82 Å), the other interactions are non-specific. Besides that, these three metabolites presented lower affinity to protein compared with piperine. This suggests that upon metabolism, the metabolites might leave the protein when in contact with other biological components.

## 3. Materials and Methods

### 3.1. Sample Preparation

A solution of piperine (100 mg, 0.35 mmol) was prepared with tetrabutylammonium hexafluorophosphate (679 mg, 1.75 mmol) (TBAPF_6_) (Sigma Aldrich^®^, St. Louis, MO, USA) (analyte: electrolyte 1:5) as the supporting electrolyte in MeCN (12 mL, Sigma Aldrich^®^).

### 3.2. Electrosynthesis of Piperine

Electrosynthesis of piperine was performed using ElectraSyn 2.0 (IKA^®^, Staufen, Germany). An undivided glass cell (electrochemical vial) equipped with a magnetic stirrer was added to the analyte solution under study. Two glassy carbon electrodes (IKA^®^, dimensions (W × H × D = 8 × 52.5 × 2 mm)), as the working electrode (WE) and the counter electrode (CE), were inserted into the solution at a distance of approximately 0.5 cm from each other. Before the experiment, the electrodes were rinsed with double distilled, deionized water, followed by the MeCN used in this study, and allowed to dry prior to the experiment. A fixed current (0.5 mA, 2.25 V maximum) was passed through the solution until the desired charge (Q) was transferred (1.33 Fmol^−1^). The electrolysis product was analyzed and monitored using TLC (SiO_2_, eluent—Toluene: EtOAc—3:2).

### 3.3. Purification and Analysis of Piperine Metabolites

Piperine metabolites were purified by flash column chromatography Biotage^®^ Isolera™ Systems (Uppsala, Sweden). DCM 100% was used to remove electrolytes from the compound mixture or by recrystallization of TBAPF_6_ using methanol, and the solvent combination (SiO_2_, eluent—DCM: isopropanol—98:2) was used to afford the title compound. The product was dissolved in CDCl_3_ (0.6 mL) with a tetramethylsilane (TMS) reference, and ^1^H and ^13^C NMR spectra were obtained.

### 3.4. Cyclic Voltammetry Procedure

All voltammetry studies were performed using an Autolab potentiostat galvanostat (PGSTAT 100 N, Utrecht, The Netherlands), and CV staircase settings were controlled by Autolab Nova 2.0 software. The CV experiments referenced ferrocene (Fc/Fc^+^) as an internal standard. An undivided glass cell (electrochemical cell) equipped with a glassy carbon electrode (GCE BASI^®^ (West Lafayette, IN, USA) MF-2012, geometric area 0.071 cm^2^ 3.0 mm diameter electrode disk of GCE material) as the working electrode, and a platinum wire (Sigma Aldrich^®^ 0.5 mm diameter) was used as a counter electrode (CE). An Ag/AgCl *pseudo* reference wire was used as the reference electrode (RE). To this electrochemical setup, the corresponding samples to be analyzed were added. The scan rates were varied using Autolab Nova 2.0 software. Before each experiment, the GCE was manually polished with 1.0-micron liquid diamond type K (Kemet, Maidstone, UK) on a smooth velvet polishing pad. The electrodes were rinsed with double-distilled, deionized water, followed by the MeCN solvent used in this study, and allowed to dry prior to the experiment. All CV data were exported to an Excel file and processed using Microsoft Excel^®^ version 16.69.1. The linear regression equations were calculated by the least square method using Microsoft Excel^®^ version 16.69.1.6. Data visualizations were presented using Prism 10.

### 3.5. Recrystallization of TBAPF_6_

The reaction mixture in MeCN was transferred into a round bottom flask, and the solvent was evaporated using a rotary evaporator. Methanol (5 mL) was added to dissolve the crude and cooled overnight in the fridge (0 °C). Crystals of TBAPF_6_ were collected either by filtration or using a Pasteur pipette to collect and separate the filtrate containing a mixture of piperine metabolites.

### 3.6. LCMS Analysis of Piperine Metabolites

Chromatographic separation was carried out using a Waters Acquity SQD2 LC-MS with UPLC consisting of a quaternary pump, autosampler, column compartment, online degasser, and diode-array detector. The chromatographical separation was conducted on an Acquity UPLC BEH C_18_ column (Waters, Milford, MA, USA; 2.1 × 50 mm, i.d., 1.7 μm) maintained at a temperature of 40 °C. The mobile phase, consisting of 0.1% formic acid in water (A) and acetonitrile (B), was delivered at a flow rate of 0.4 mL/min. The gradient elution program was optimized as follows: 15% B at 0–1 min, 15–30% B at 1–5 min, 30–55% B at 5–10 min, 55–90% B at 10–13 min, and 15% B at 13–15 min. The diode-array detector was set at a range of 190–400 nm. Mass detection was carried out on a Waters SQD2 electrospray ionization, single quadrupole mass spectrometer equipped with positive and negative electrospray ionization (ESI) sources. The source conditions were optimized as follows: spray voltage, 3.0 kV; sheath gas (N_2_) flow rate, 30 arbitrary units (arb); auxiliary gas (N_2_) flow rate, 10 arb; and capillary temperature, 300 °C. Full mass spectra were recorded from *m*/*z* 120 to 750 in centroid mode. All the operations and the post-data processing were controlled by MassLynx 4.1 SCN855 software.

### 3.7. Docking Procedures

The molecular piperine and metabolite structures were optimized by ab initio calculation. The calculations were performed using the Gamess2018 quantum mechanics package with Hartree–Fock (HF) formalism and functional density theory (DFT) following the same method as our previous work [42]. Briefly, 6-31+G (d, p) was used as the first set of bases followed by a structure refinement with the set of bases 6-311+G(2d,2p) and B3LYP functional. The protein structure was obtained from PDB 1TQN and prepared following the same method as our previous work [38]. AutoDock tools 1.5.4 was used to prepare the protein, adding polar hydrogen bonds and Gasteiger charges. A grid box was built to explore the whole protein (blind docking) with the grid box dimensions as 126 × 126 × 126 points with a spacing of 0.458 Å and centered at x = −19.213, y = −23.825, and z = −14.03. The protein binding sites were investigated with autodock4.2 using the Lamarckian Genetic Algorithm (LGA) in a total of 100 different conformations. The final poses were selected among the most negative energies. AutoDock tools 1.5.4 was used for pi–pi stacking analysis.

## 4. Conclusions

In summary, we have disclosed a direct electrochemical analysis and reaction of piperine and identified a series of chemically plausible metabolites. Furthermore, predictive modeling of piperine identified the most likely region of the molecule to undergo oxidation in the body. Analysis of the piperine metabolite binding pose within the active heme Fe pocket of CYP3A4 revealed potential molecules that may lead to the inhibitive activity of piperine reported. Taken together, we have shown an approach that integrates computational docking, electrochemical reaction, and analytical techniques to predict the likelihood of metabolites in a challenging example. The CP-EDM technique may find use in a range of drug discovery endeavors to expedite the prediction and analytical aspects of drug metabolism.

## Figures and Tables

**Table 1:** Scan rate study results analysis of the initial piperine oxidation wave (Epa1).

Scan Rate (Vs^−1^)	Anodic Peak Potential Ep_1_ (mV)	Anodic Peak Current Ip_1_ (µA)	Square Root of Scan Rate v^1/2^ (V/s)^1/2^	Diffusion Coefficient (10^−6^ cm^2^ s^−1^)
0.05	783	49	0.224	1.0312
0.10	791	71	0.316	1.0825
0.15	811	86	0.387	1.0588
0.20	812	104	0.447	1.1613
0.25	825	114	0.500	1.1163
0.30	832	124	0.548	1.1006

**Table 2:** Plot of data from scan rate and concentration studies.

No	Data Plot	Linear Regression
1	Scan rate (mVs^−1^) vs. peak current (Ip_1_)	Y = 0.2983 x + 39.13 R^2^ = 0.9792
2	Square root of scan rate v^1/2^ (V/s)^1/2^ vs. peak current (Ip_1_) (µA)	Y = 234.3 x − 3.248 R^2^ = 0.9975
3	Log of scan rate (mVs^−1^) vs. Log of peak potential (Ep_1_) mV	Y = 0.03439 x + 2.833 R^2^ = 0.9401
4	Log of scan rate (mVs^−1^) vs. Log of peak current (Ip_1_) (µA)	Y = 0.5230 x + 0.8030 R^2^ = 0.9983
5	Scan rate (mVs^−1^) vs. peak potential (Ep_1_) mV	Y = 0.1989 x + 774.2 R^2^ = 0.9983
6	Concentration vs. peak current (Ip_1_) (µA)	Y = 18.13 x + 32.80 R^2^ = 0.9585
7	Concentration vs. peak potential (Ep_1_) mV	Y = 11.07 x + 767.8 R^2^ = 0.7492

**Table 3:** The comparison with previously detected piperine metabolites from tandem electrochemical–LCMS detection [26], hepatocyte incubation [27], and this work. Codes in columns 2 and 3, e.g., C# and M#, are directly taken from the original literature assignment codes [26,27].

Predicted Oxidative Piperine Metabolites	Electrochemical Azam et al. [26]	Hepatocytes Li et al. [27]	This Work
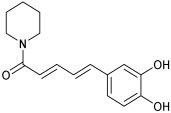	C12	M12	n.d.
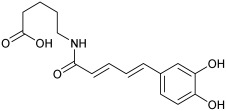	C4	n.d.	n.d.
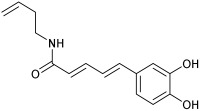	C8	M8	n.d.
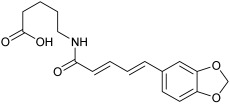	n.d.	M14	n.d.
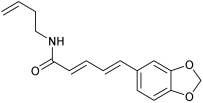	n.d.	M19	M5-1
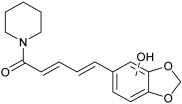	n.d.	M18	n.d.
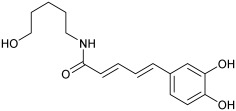	n.d.	M3	n.d.
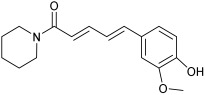	C17	n.d.	M6
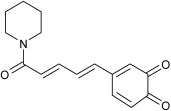	GSH-adduct Detected via *ortho*-quinone	n.d.	M5-2
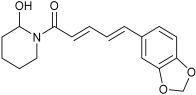	n.d.	n.d.	M2
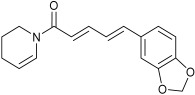	n.d.	n.d.	M3

“n.d.”—not detected.

## Data Availability

The original contributions presented in this study are included in this article and Supplementary Materials. Further inquiries can be directed to the corresponding authors.

## Supplementary Materials

The following supporting information can be downloaded at https://www.mdpi.com/article/10.3390/molecules29102406/s1, Figure S1: Visualization of two docking poses (M5-1 and M5-2) highlighting the hydrogen bond interaction, Figure S2: The clusters of molecular docking calculation, Table S1: Binding energy score of molecular docking, procedures, LCMS spectra of metabolites, piperine standard analysis, ^1^H NMR spectra of isolated #1 spot, LCMS spectrum of isolated #1 spot, and CV studies of piperine, and reference [42].
