# Supplementary material for: Computational Predictive and Electrochemical Detection of Metabolites (CP-EDM) of Piperine
Source: Molecules. 2024 May 20;29(10):2406. doi: 10.3390/molecules29102406 (PMC11123718; doi:10.3390/molecules29102406)
Supplement: Supplementary file 1 [file molecules-29-02406-s001.zip › molecules-2996565-supplementary.pdf]

SUPPLEMENTARY INFORMATION

Computational Predictive and Electrochemical Detection of Metabolites (CP-EDM) of Piperine

Ridho Asra 1, Ana P. R. Povinelli 2, Gabriel Zazeri 3,* and Alan M. Jones 1,*

1 School of Pharmacy, College of Medical and Dental Sciences, University of Birmingham, Birmingham B15 2TT, UK; rxa240@student.bham.ac.uk
2 Departament of Physics, Instituto de Biociências, Letras e Ciências Exatas (IBILCE), UNESP, Rua Cristovão Colombo 2265, São José do Rio Preto 15054-000, SP, Brazil; anapovinelli@outlook.com
3 Departament of Physics, Universidade Federal de Roraima (UFRR). Av. Cap. Ene Garcês, 2413—Aeroporto, Boa Vista 69310-000, RR, Brazil
* Correspondence: gabriel.zazeri@ufrr.br (G.Z.); a.m.jones.2@bham.ac.uk (A.M.J.)

CONTENTS

ITEM	PAGE #
**Figure S1**	**2**
**Figure S2**	**3**
**Table S1**	**4**
**Procedures**	**5-6**
**LCMS spectra of all metabolites detected**	**7-22**
**Piperine standard analysis**	**23-24**
**^{1}H NMR spectra of isolated #1 spot**	**25**
**LCMS spectra of isolated #1 spot**	**26-28**
**CV studies of piperine**	**29-32**
**References**	**33**

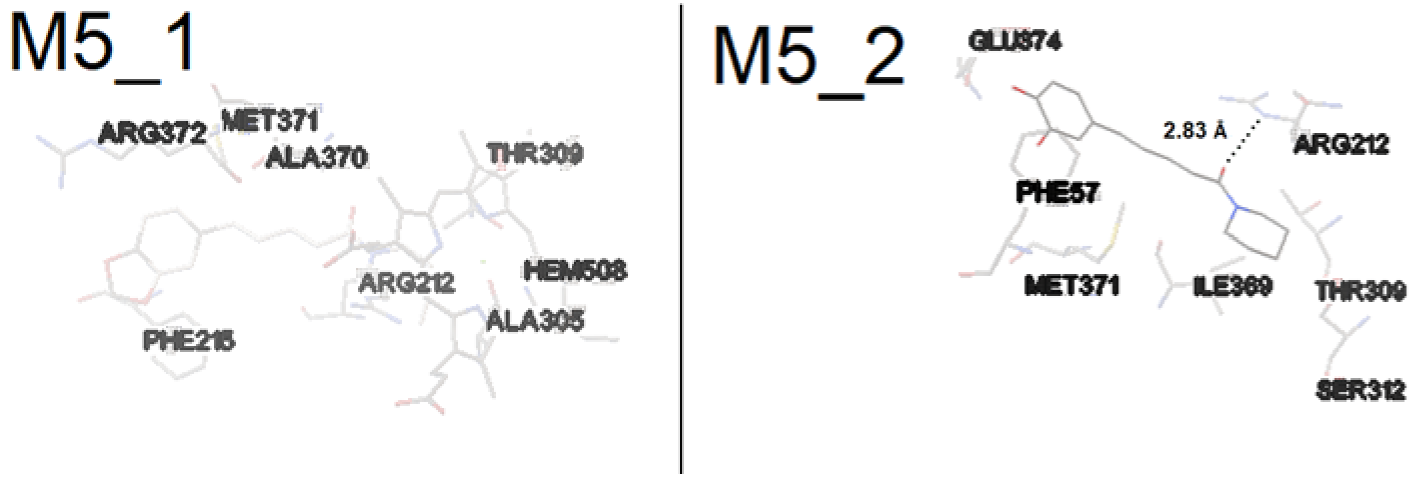

Figure S1. The visualization of the two docking poses (**M5-1** and **M5-2**) highlighting the hydrogen bond interaction.

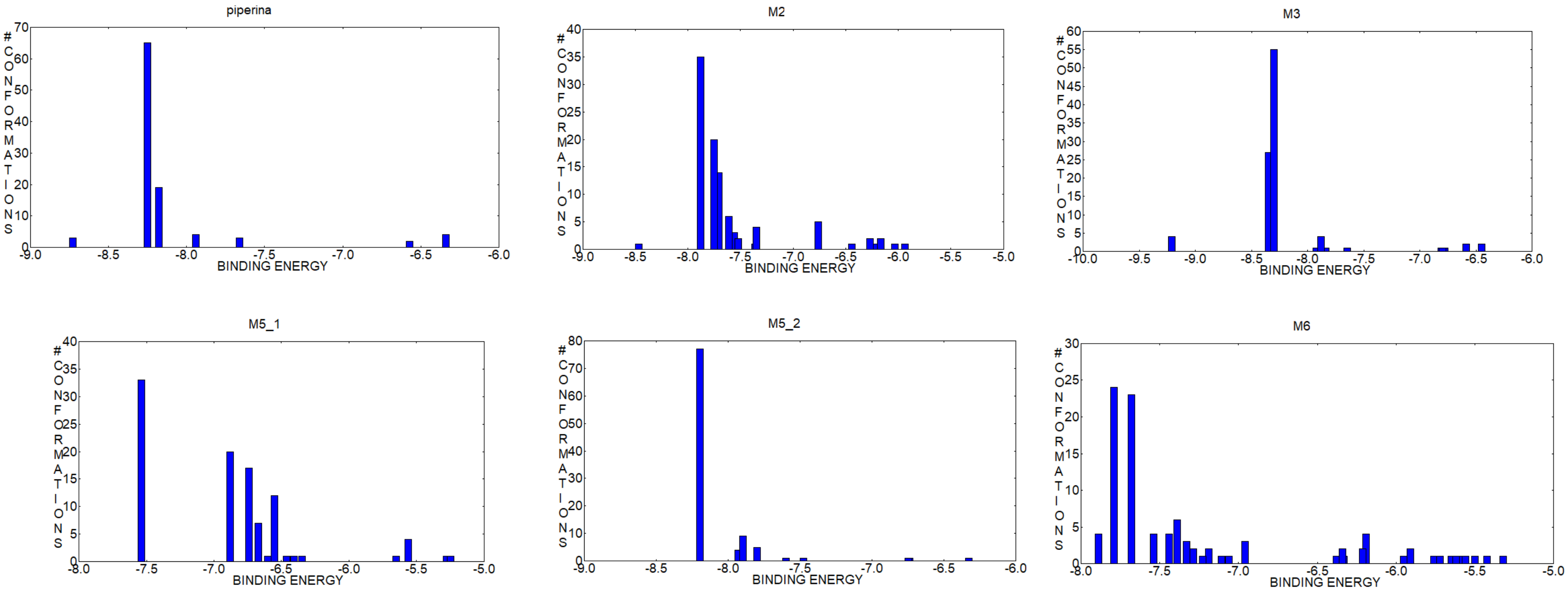

Figure S2: The clusters of molecular docking calculation.

Molecule	Binding energy score (kcal/mol)
**Piperine**	-8.73
**M2**	-8.47
**M3**	-9.21
**M5-1**	-7.54
**M5-2**	-8.2
**M6**	-7.89

Table S1: Binding energy score os molecular docking

.

Procedures

1.1. *Sample preparation*

A solution of 100 mg (0.35 mmol) piperine was prepared in a beaker containing 679 mg (1.75 mmol) tetrabutylammonium hexafluorophosphate ($TBAPF_6$) (Sigma Aldrich®) (analyte: electrolyte 1:5) as the supporting electrolyte in 12 mL MeCN (Sigma Aldrich®).

1.2. *Electrosynthesis of Piperine*

Electrosynthesis of piperine was performed using ElectraSyn 2.0 (IKA®). An undivided glass cell (electrochemical vial) equipped with a magnetic stirrer was added analyte solution under study. Two glassy carbon electrodes (IKA®, Dimensions (W x H x D = 8 x 52.5 x 2 mm) as the working electrode (WE) and the counter electrode (CE) were inserted into the solution at a distance of approximately 0.5 cm from each other. Before the experiment, the electrodes were rinsed with double distilled, deionised water, followed by MeCN used in this study, and allowed to dry prior to the experiment. A fixed current (0.5 mA, 2.25 V maximum) was passed through the solution until the desired charge (Q) was transferred (1.33 F/mol). The electrolysis product was analysed and monitored using TLC (SiO2, eluent - Toluene: EtOAc – 3:2).

1.3. Purification and analysis of piperine metabolites

A piperine metabolite was purified by flash column chromatography Biotage® Isolera™ Systems. DCM 100% was used to remove electrolytes from the compound mixture or by using recrystallisation of $TBAPF_6$ using methanol, and the solvent combination (SiO_2, eluent - DCM: isopropanol – 98:2) was used to afford the title compound. The product was dissolved in $CDCl_3$ (0.6 mL) with a TMS reference, and ^1H and ^{13}C NMR spectra were obtained.

1.4 Cyclic Voltammetry Procedure

All voltammetry studies were performed using an Autolab potentiostat galvanostat (PGSTAT 100 N, The Netherlands), and CV staircase settings were controlled by the Autolab Nova 2.0 software. The CV experiments referenced ferrocene (Fc/Fc+) as an internal standard. An undivided glass cell (electrochemical cell) equipped with a glassy carbon electrode (GCE BASI® MF-2012, geometric area 0.071 cm^2 3.0 mm diameter electrode disk of GCE material) as the working electrode, a platinum wire (Sigma Aldrich® 0.5 mm diameter) was used as a counter electrode (CE). Ag/AgCl pseudo reference wire was used as the reference electrode (RE). To this electrochemical set-up were added the corresponding samples to be analysed. Scan rates were varied using the Autolab Nova 2.0 software. Before each experiment, the GCE was manually polished with 1.0-micron liquid diamond type K (Kemet, Maidstone, UK) on a smooth velvet polishing pad. The electrodes were rinsed with double distilled, deionised water, followed by suitable solvents used in this study, and allowed to dry prior to the experiment. All CV data were exported to an Excel file and processed using Microsoft Excel® version 16.69.1. The linear regression equations were calculated by the least square method using Microsoft Excel® version 16.69.1.

1.5 Recrystallisation of $TBAPF_6$

The reaction mixture in MeCN was decanted into the flask, and MeCN was evaporated using a rotary evaporator. Methanol (5 mL) was added to dissolve the crude and cooled overnight in the fridge (0 °C). The crystal of $TBAPF_6$ was collected either by filtration or using a chemical dropper to collect and separate the filtrate containing a mixture product of piperine metabolites.

1.6 LCMS analysis of piperine metabolites

Chromatographic separation was carried out using a Waters Acquity SQD2 LC-MS with UPLC consisting of a quaternary pump, autosampler, column compartment, online degasser, and diode-array detector. The chromatographical separation was conducted on an Acquity UPLC BEH C_{18} column (Waters, Milford, MA, USA; 2.1 × 50 mm, i.d., 1.7 μm) maintained at a temperature of 40 °C. The mobile phase, consisting of 0.1% FA in water (A) and acetonitrile (B), was delivered at a flow rate of 0.4 mL/min. The gradient elution program was optimised as follows: 15% B at 0–1min, 15%–30% B at 1–5 min, 30%–55% B at 5–11 min, 55%–90% B at 11–15 min, and 15% B at 15–17 min (20 minutes run time). The diode-array detector was set at a range of 190–400 nm. The mass detection was carried out on a Waters SQD2 electrospray ionisation, single quadrupole mass spectrometer equipped with positive and negative electrospray ionisation (ESI) sources. The source conditions were optimised as follows: spray voltage, 3.0 kV; sheath gas (N2) flow rate, 30 arbitrary units (arb); auxiliary gas (N_2) flow rate, 10 arb; capillary temperature, 300 °C. Full mass spectra were recorded from m/z 120 to 750 in centroid mode. All the operations and the post-data processing were controlled by MassLynx 4.1 SCN855 software.

1.7 Docking procedures

The molecular piperine and metabolite structures were optimized by *ab initio* calculation. The calculations were performed using the Gamess2018 quantum mechanics package with Hartree–Fock (HF) formalism and functional density theory (DFT) following the same method of our previous work. [S1] The protein structure was obtained from PDB 1TQN and prepared following the same method of our previous work (add reference https://doi.org/10.1016/j.molstruc.2021.130995) . Autodock tools 1.5.4 were used to prepare the protein, adding polar hydrogen bonds and Gasteiger charges. The Grid box was built to explore the whole protein (blind docking) with the grid box dimensions as 126×126×126 points with a spacing of 0.458 A˚ and centered at x = -19.213, y =-23.825, and z = -14.03. The protein binding sites were investigated with autodock4.2 using the Lamarckian Genetic Algorithm (LGA) in a total of 100 different conformations. The final poses were selected among the most negative energies

LCMS Analysis

Reaction mixture post-electrochemical reaction

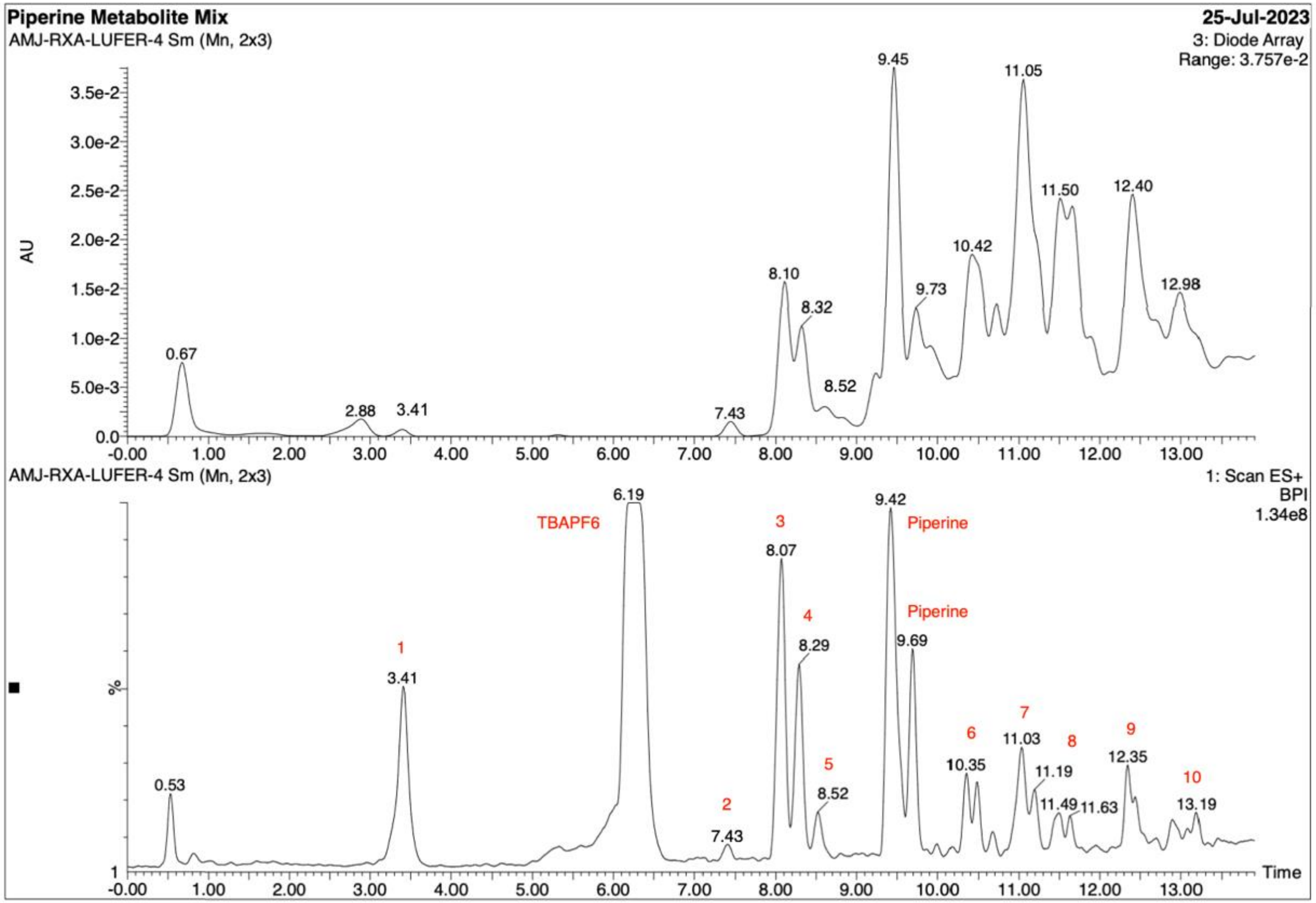

Compound	Peak	Retention time (min)	Elemental position	Measured m/z	AMU*	Calculated m/z	Structure*
M1	1	3.41	?	186.35	185.34	?	?
TBA	E	6.19	$C_{16}H_{36}N^{+}$	242.55	242.55	242.47	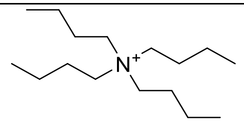 Chemical Formula: $C_{16}H_{36}N^{+}$<br>Molecular Weight: 242.47
M2	2	7.32	$C_{17}H_{19}NO_4$	302.53	301.52	301.34	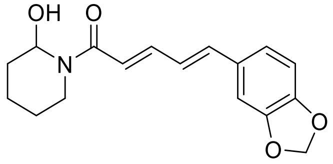 Chemical Formula: $C_{17}H_{19}NO_4$<br>Molecular Weight: 301.34
M3		7.41	$C_{17}H_{18}NO_3^{+}$	284.38	284.38	284.33	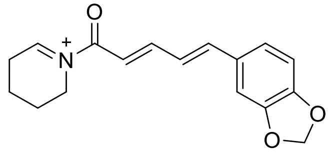 Chemical Formula: $C_{17}H_{18}NO_3^{+}$<br>Molecular Weight: 284.33<br>or
			$C_{17}H_{17}NO_3$			283.33	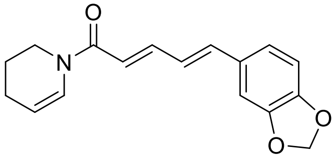 Chemical Formula: $C_{17}H_{17}NO_3$<br>Molecular Weight: 283.33

M4		7.45	?	356.40	355.39	?	?
M5	3<br>4<br>5	8.09<br>8.28<br>8.55	$C_{16}H_{17}NO_3$	272.35<br>272.35<br>272.29	271.34<br>271.34<br>271.28	271.32	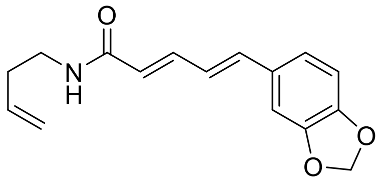<br>Chemical Formula: $C_{16}H_{17}NO_3$<br>Molecular Weight: 271.32<br>Or<br>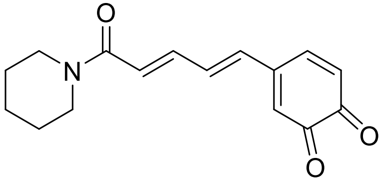<br>Chemical Formula: $C_{16}H_{17}NO_3$<br>Molecular Weight: 271.32
M6	S	9.23	$C_{17}H_{21}NO_3$	288.42	287.41	287.15	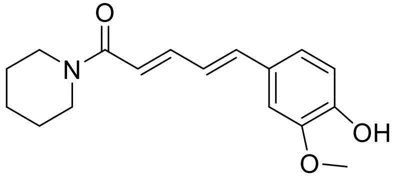<br>Chemical Formula: $C_{17}H_{21}NO_3$<br>Molecular Weight: 287.36
Piperine<br>Piperine		9.48<br>9.53	$C_{17}H_{19}NO_3$<br>$C_{17}H_{19}NO_3$	286.40<br>286.34	285.39<br>285.33	285.44<br>285.34	

							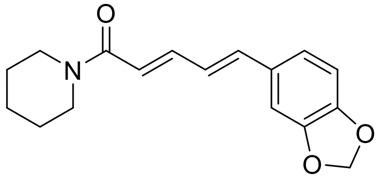<br>Chemical Formula: $C_{17}H_{19}NO_3$<br>Molecular Weight: 285.34
M7	6	10.34	?	518.53	517.52	?	?
M8	7	11.05	?	502.52	501.51	?	?
M9	8	11.44	?	472.53	471.52	?	?
M10		11.62	?	502.52	501.51	?	?
M11	9	12.34	?	516.57	515.56	?	?
M12		12.89	?	569.63	568.62	?	?
M13	10	13.07	?	569.63	568.62	?	?
M14		13.19	?	601.64	600.63	?	?

*LCMS ES^+: actual molecular weight (amu) = MW from LCMS ES^+ (amu) - Mass of proton (1.0073 amu), Structures were generated from ChemDraw 20.0.0.41

Background Check (Water)

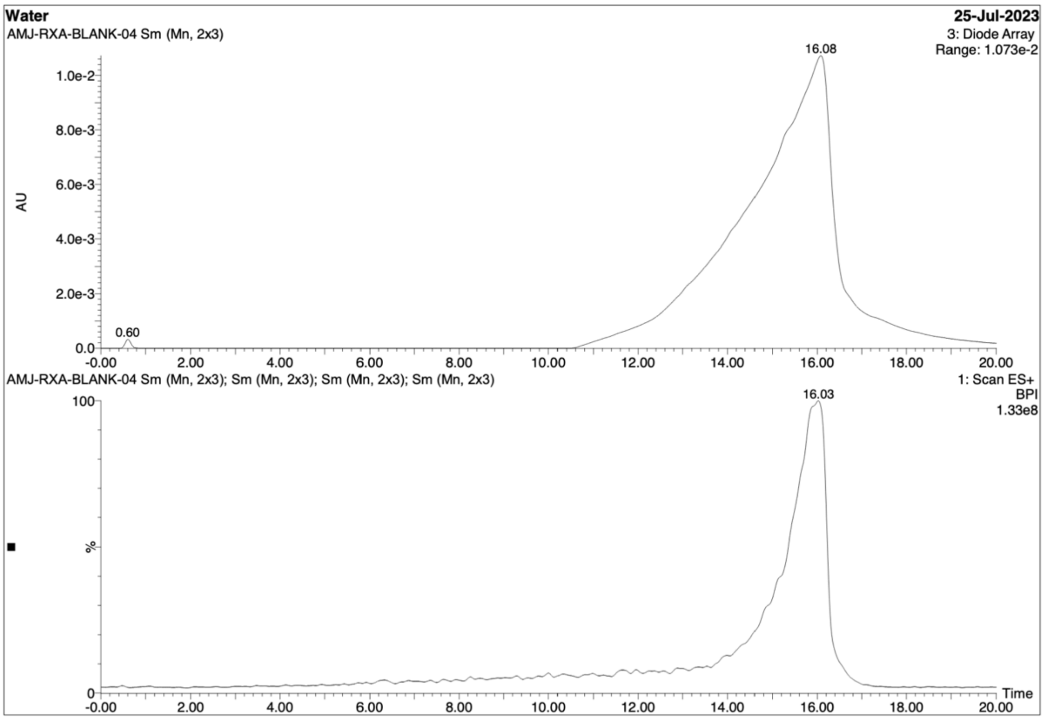

Piperine (standard)

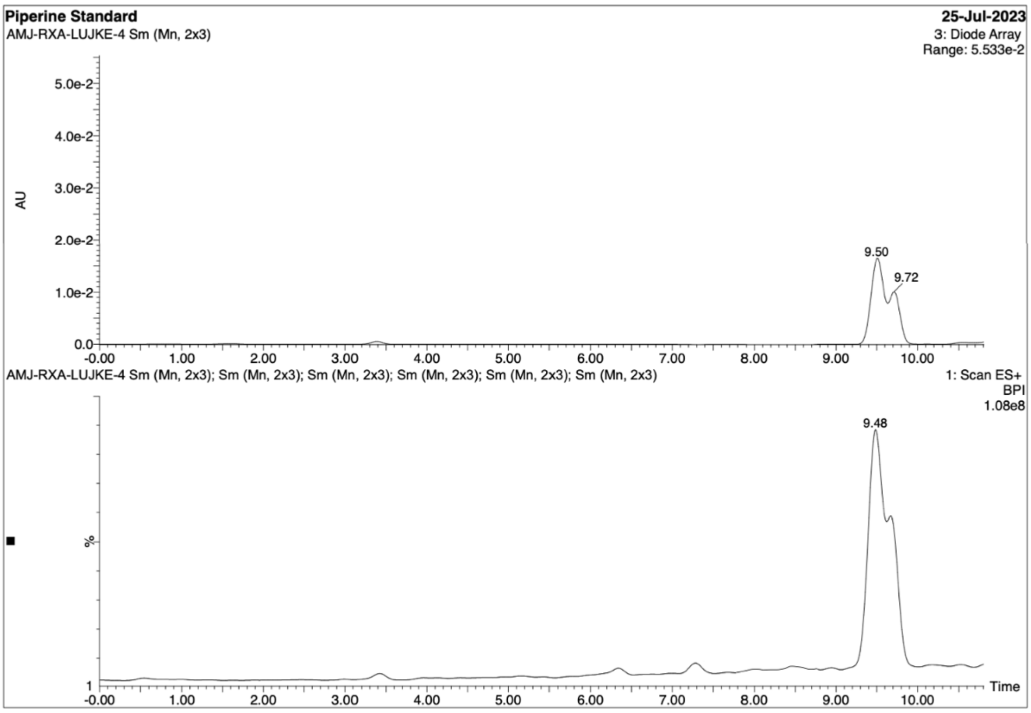

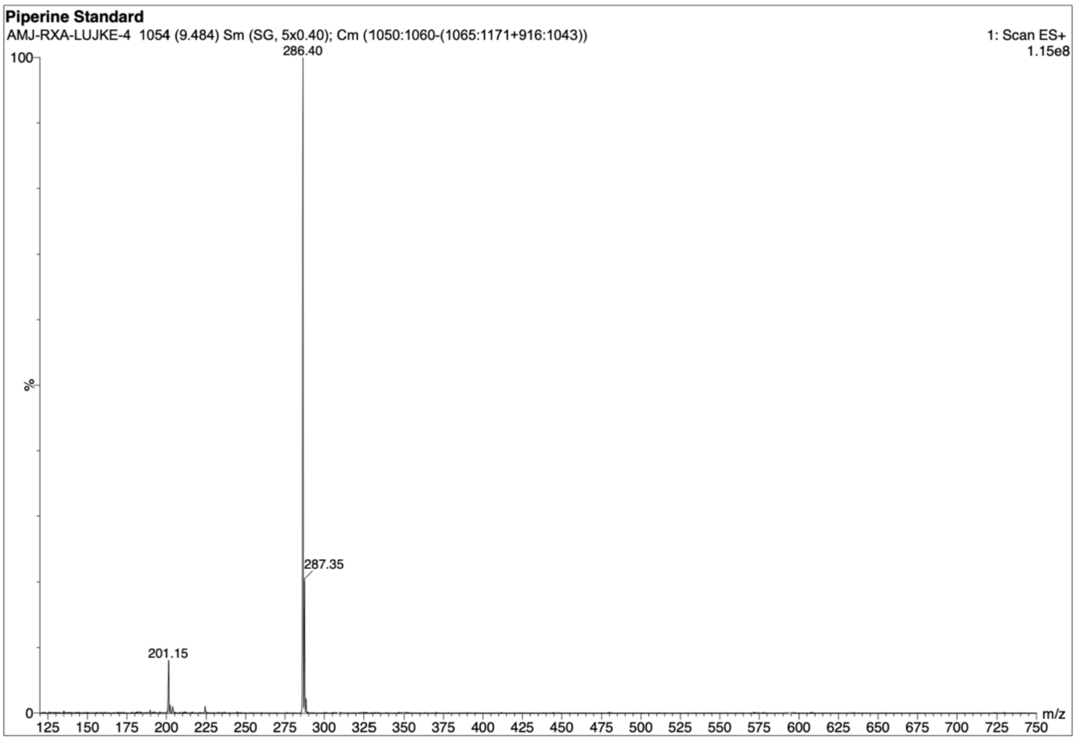

Isolated piperine metabolite (impure)

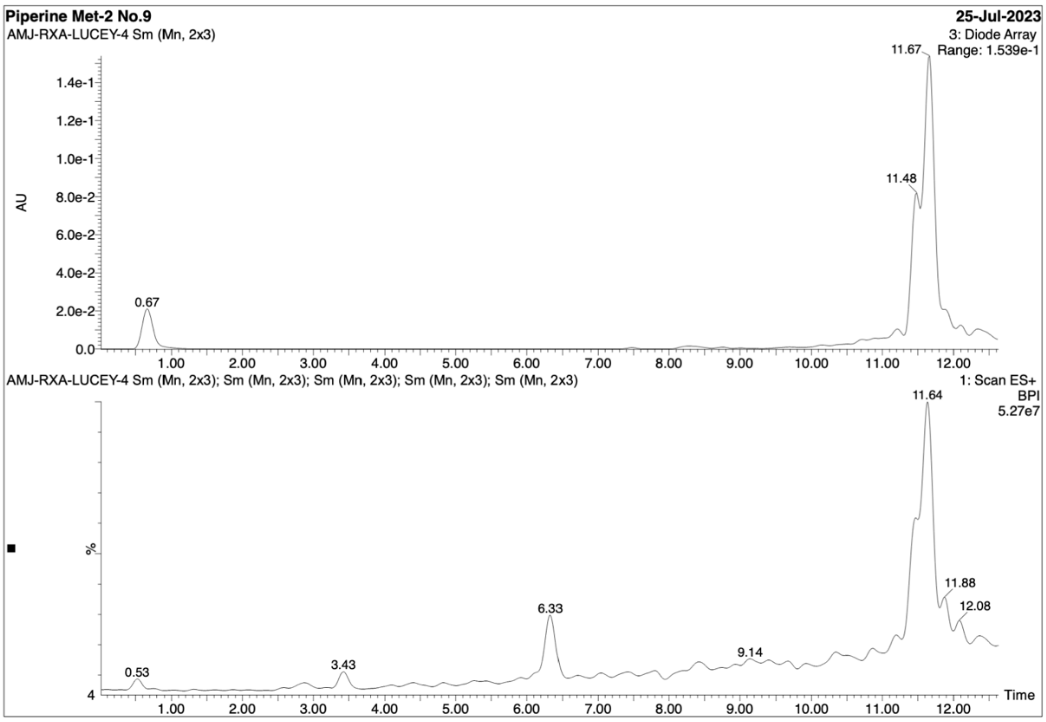

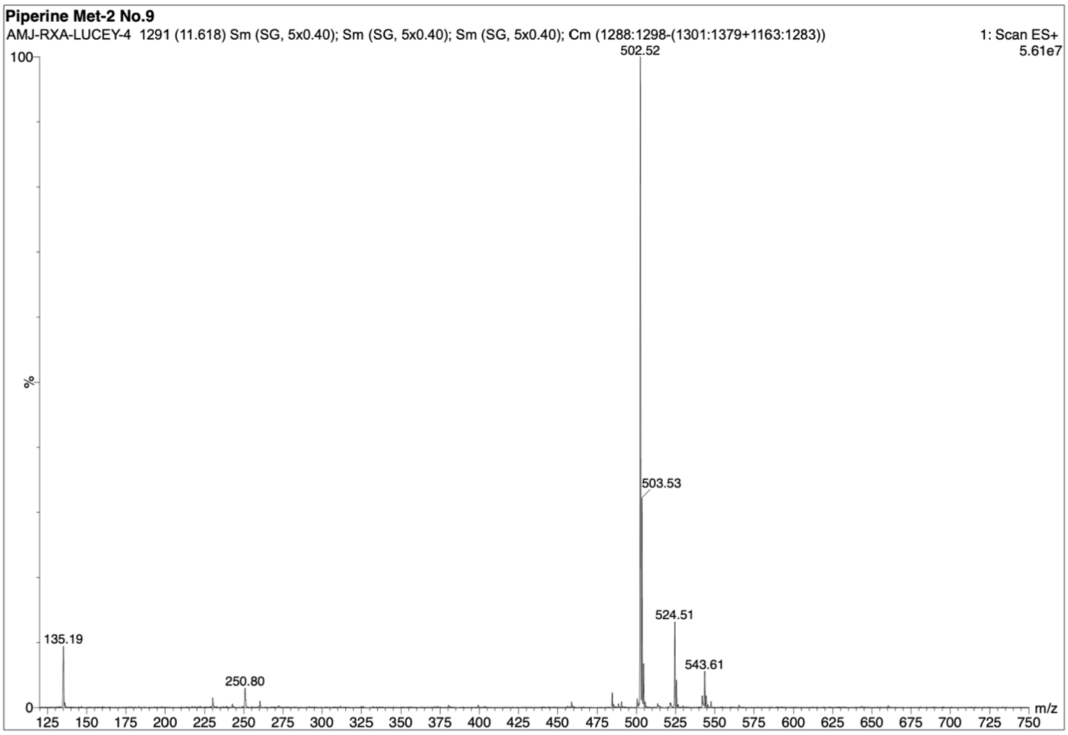

Metabolite 1

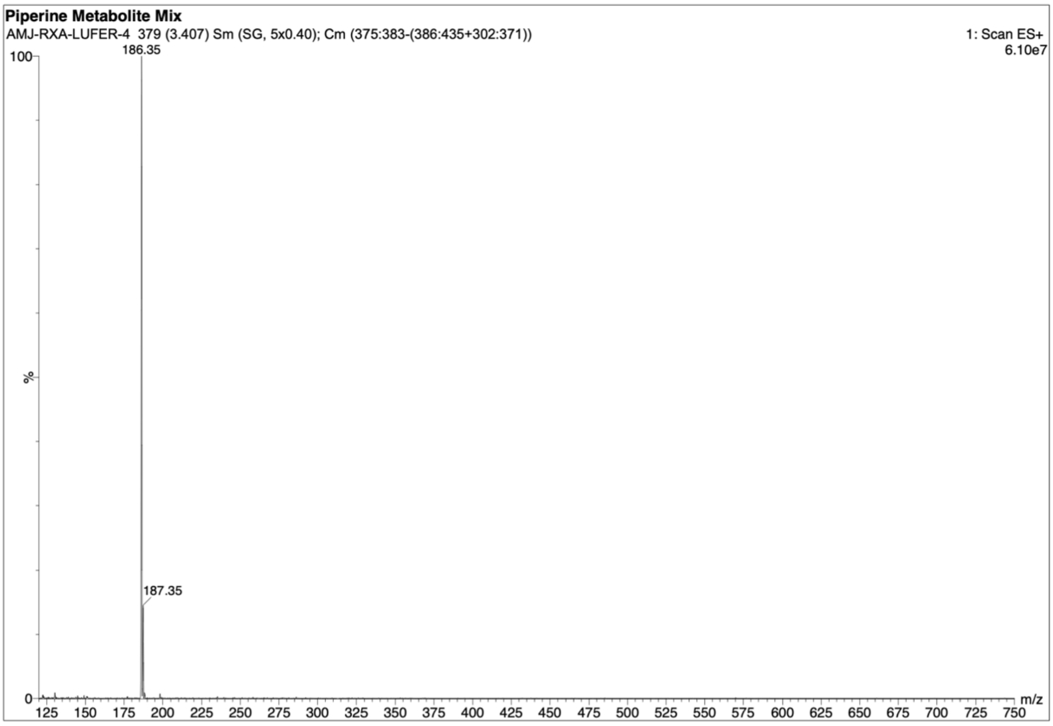

TBA

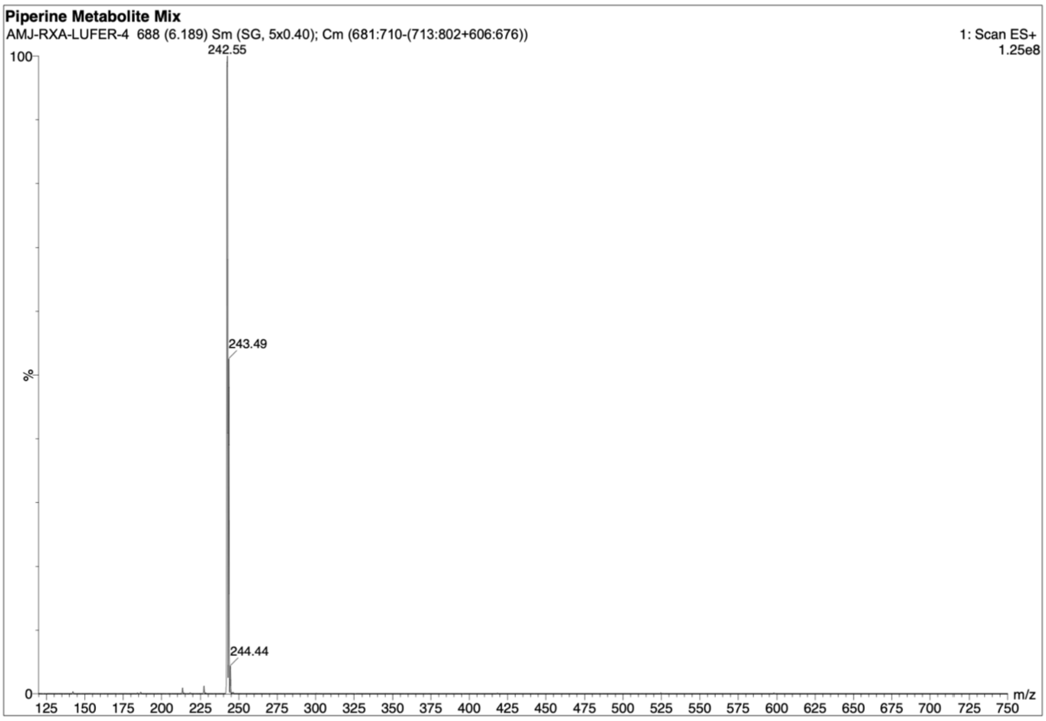

Metabolite 2

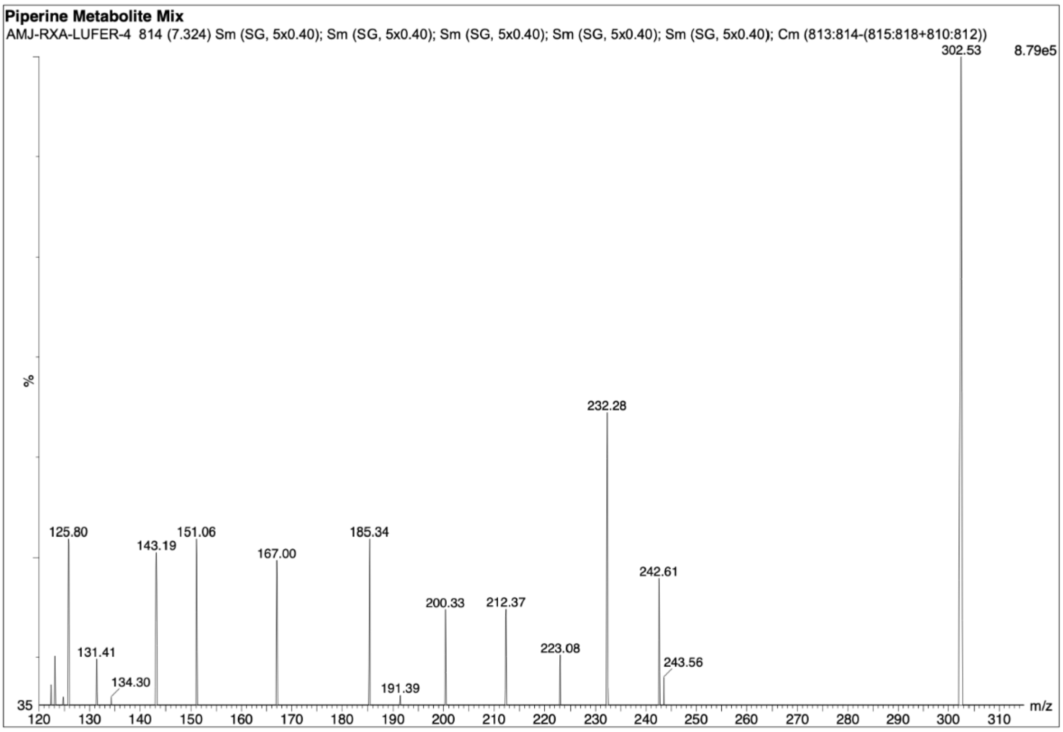

Metabolite 3

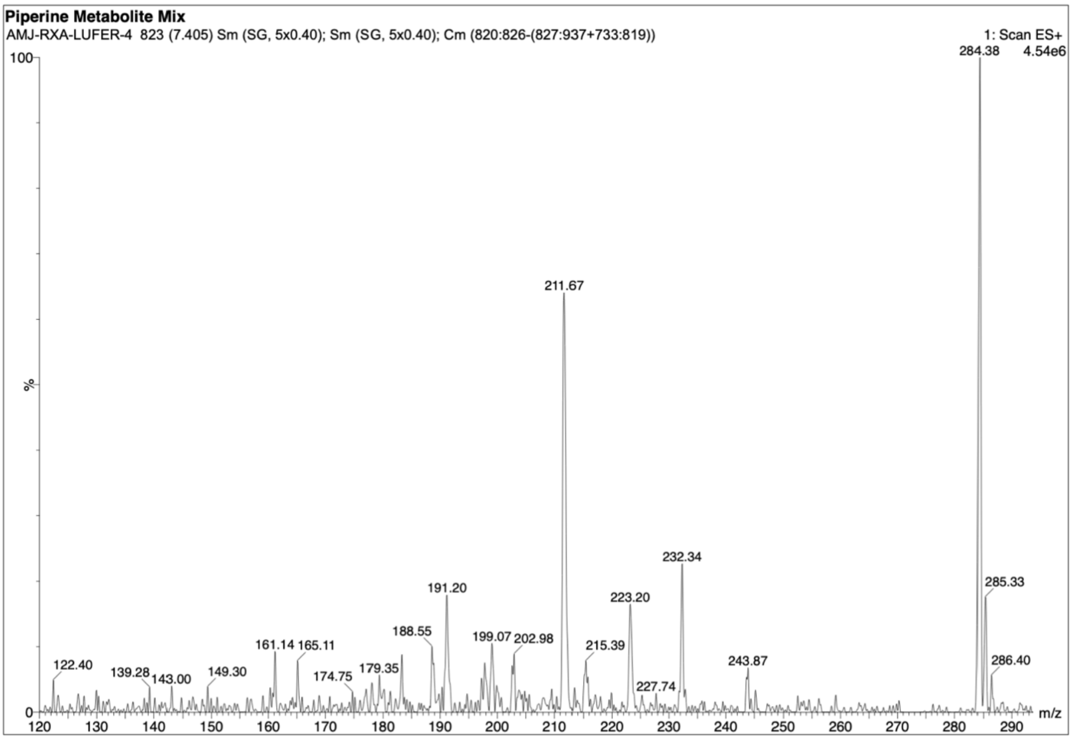

Metabolite 4

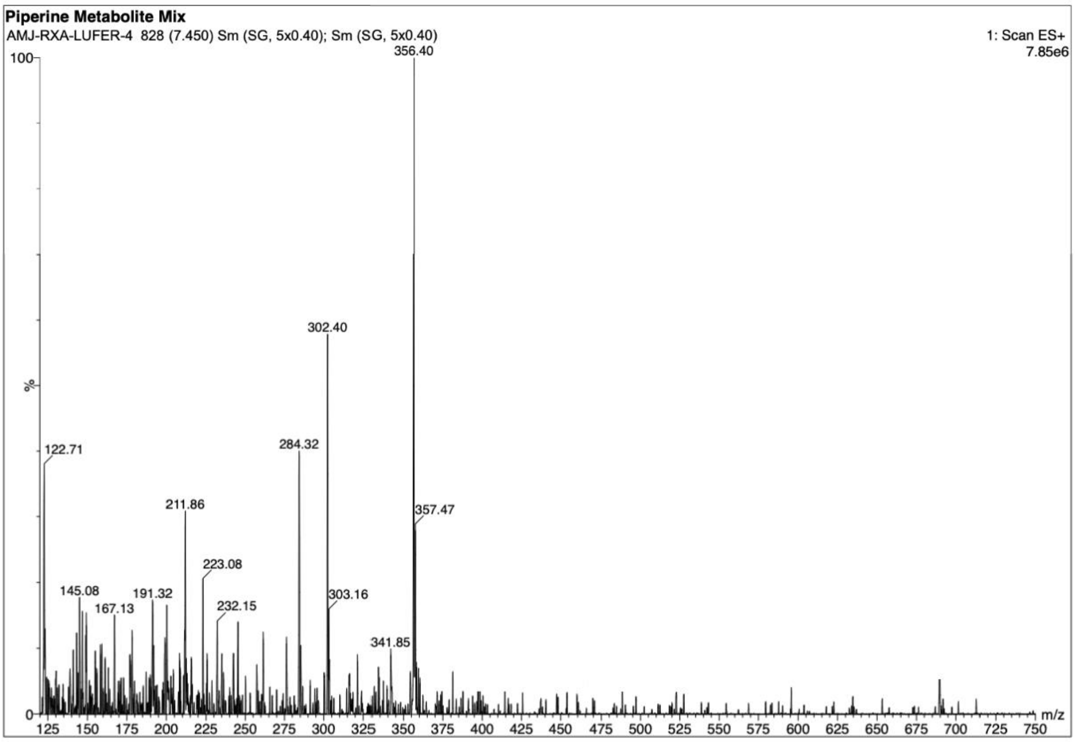

Metabolite 5-1

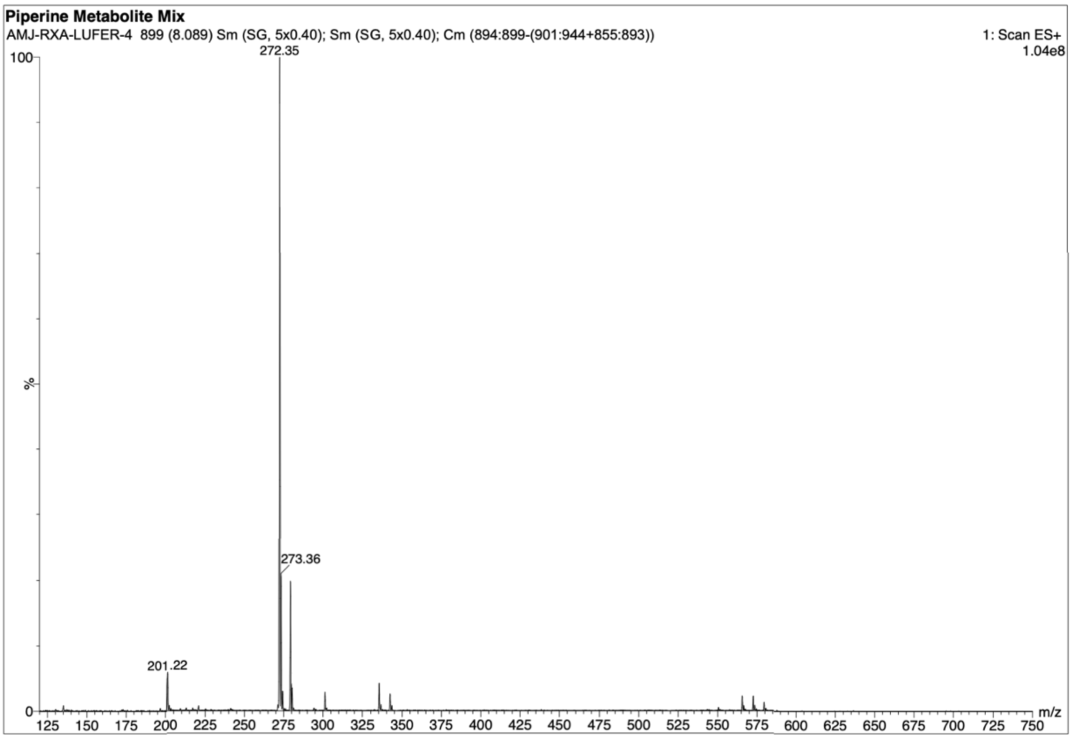

Metabolite 5-2

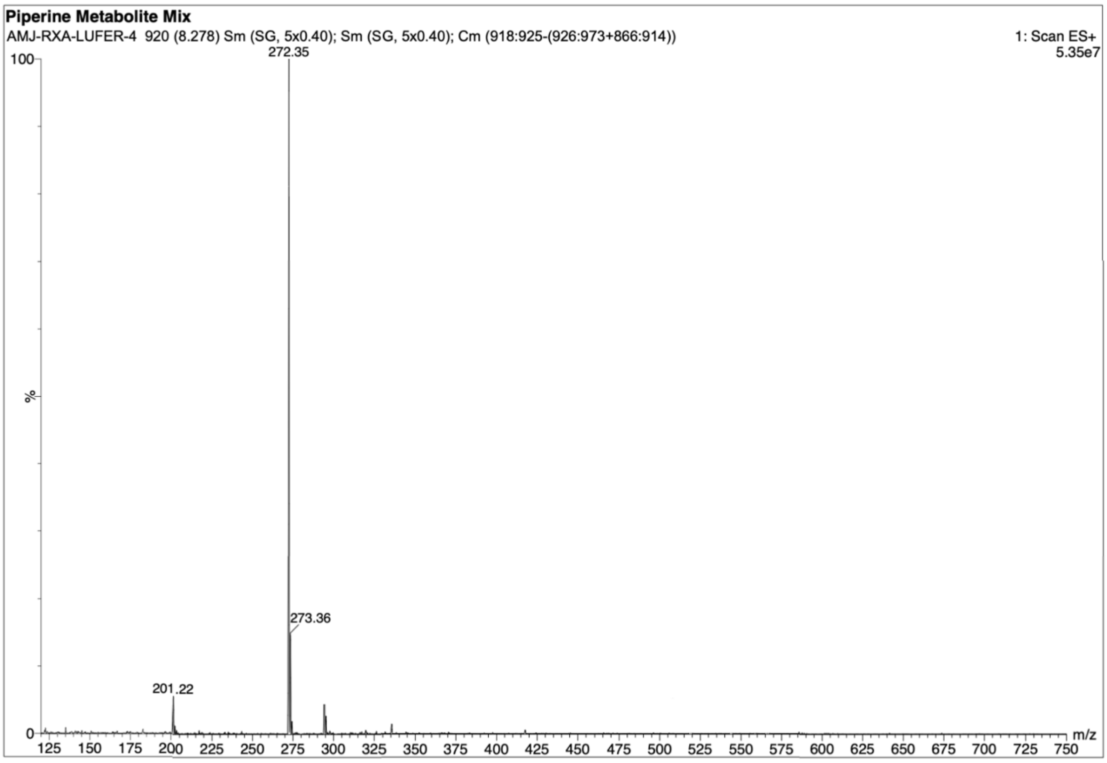

Metabolite 5-3

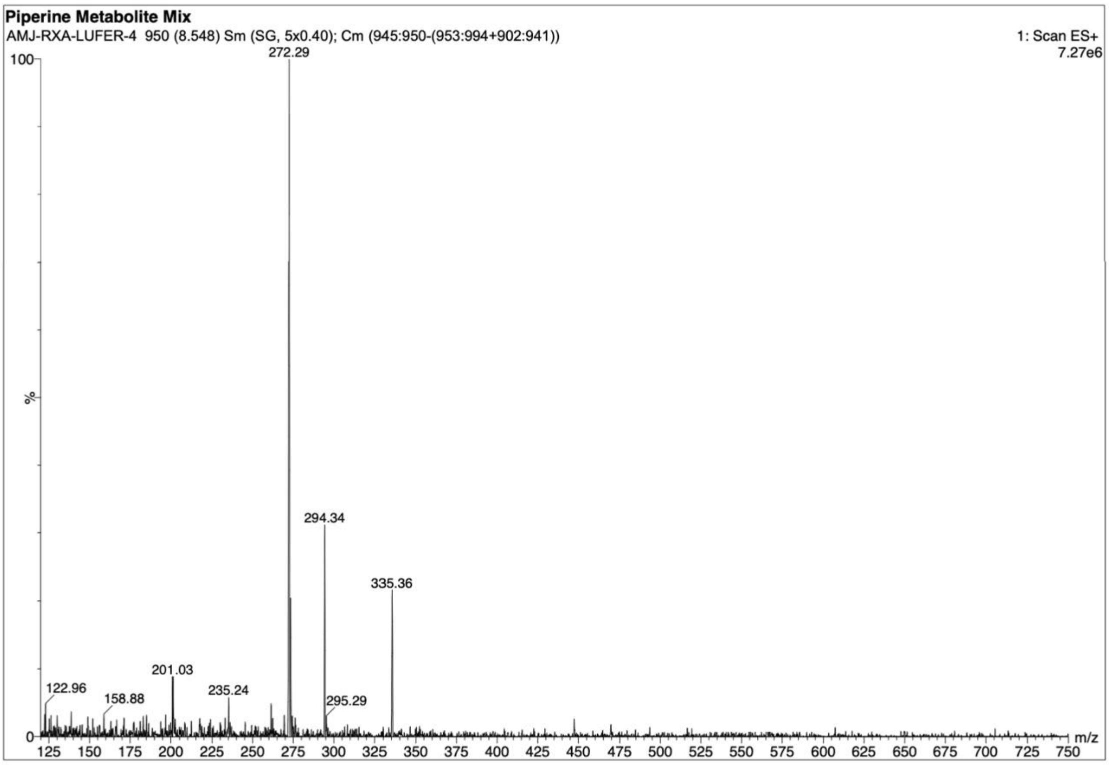

Metabolite 6

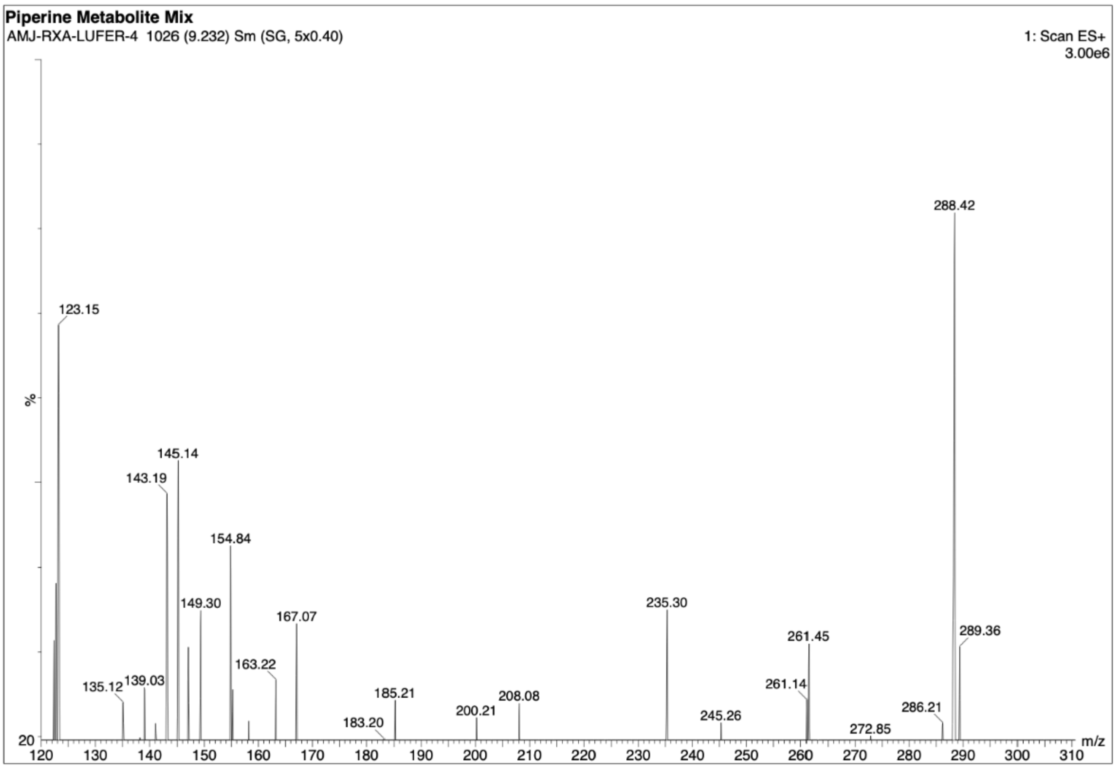

Piperine 1-1

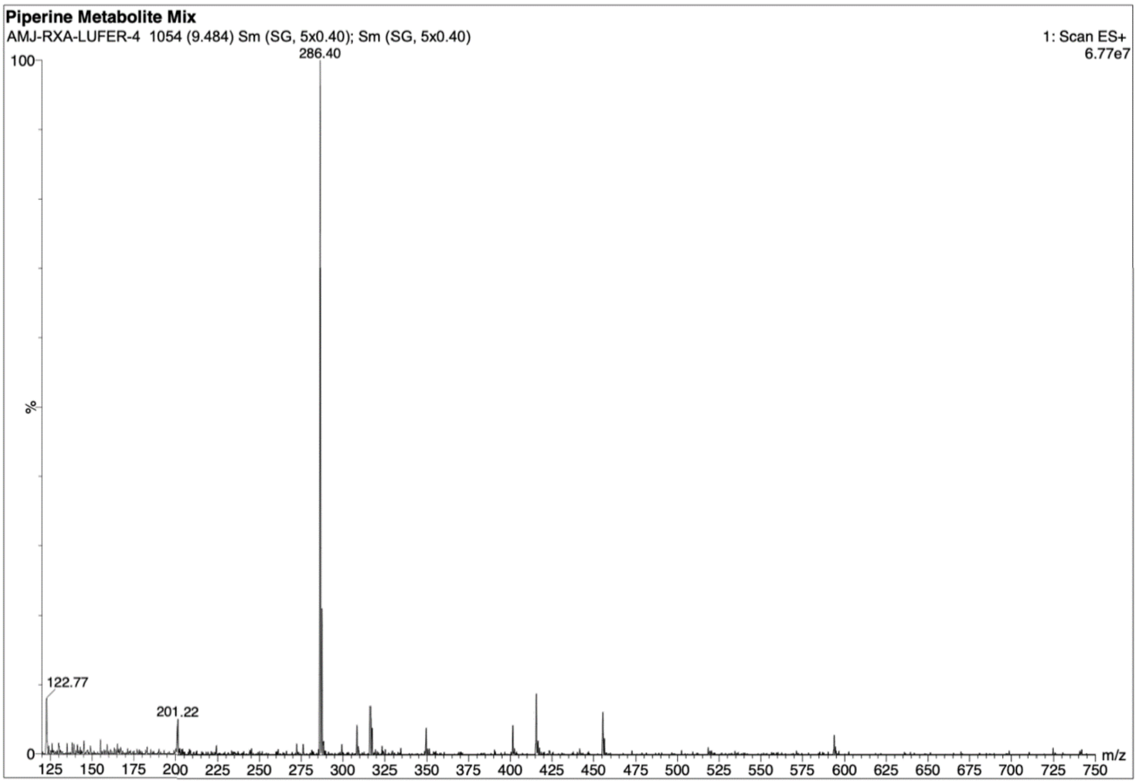

Piperine 1-2

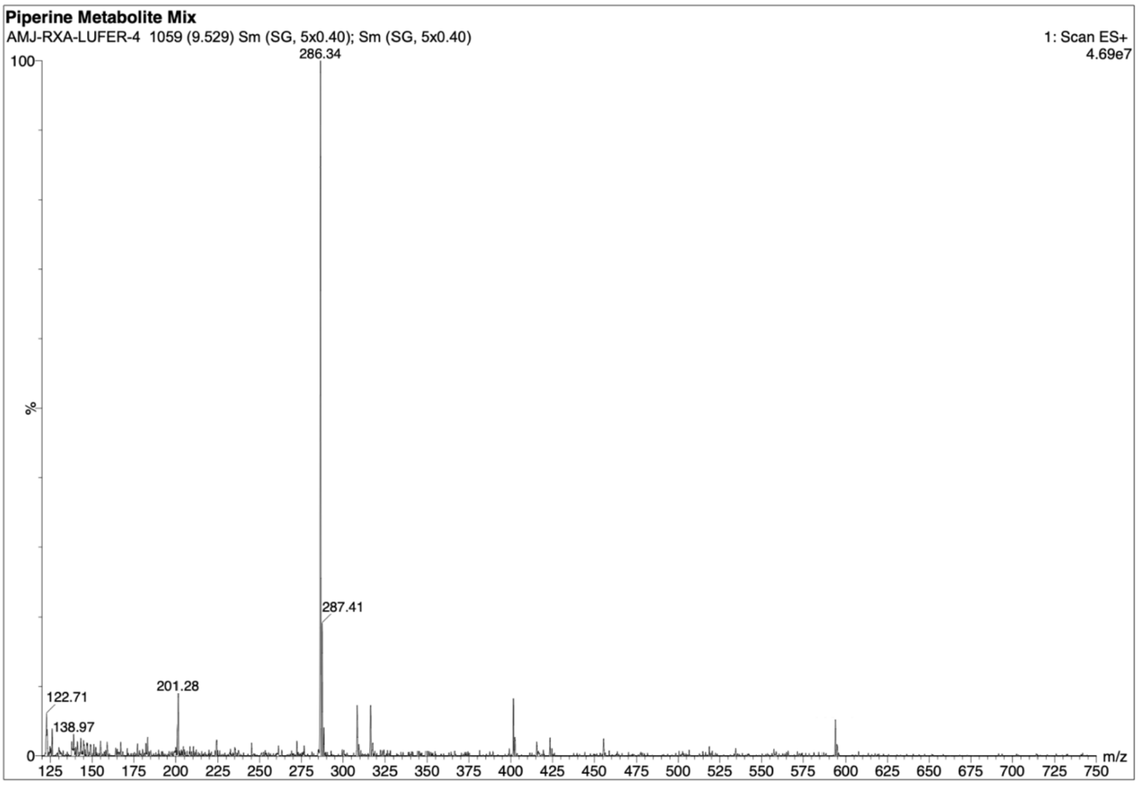

Metabolite 7

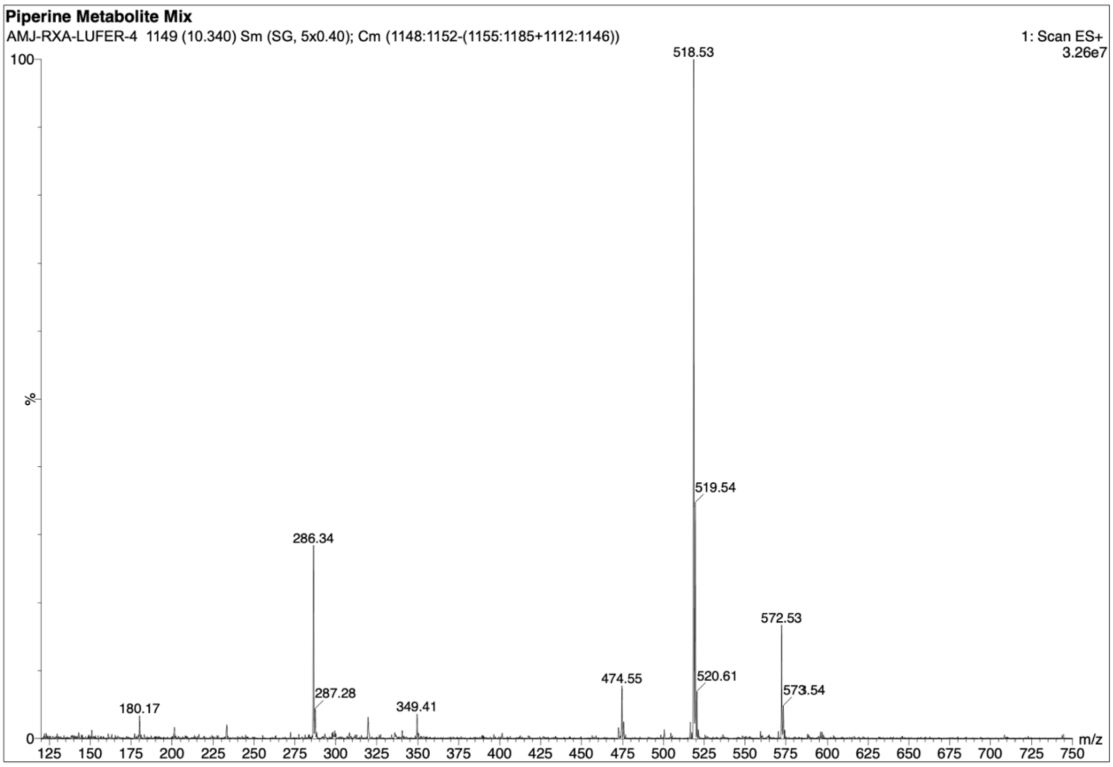

Metabolite 8

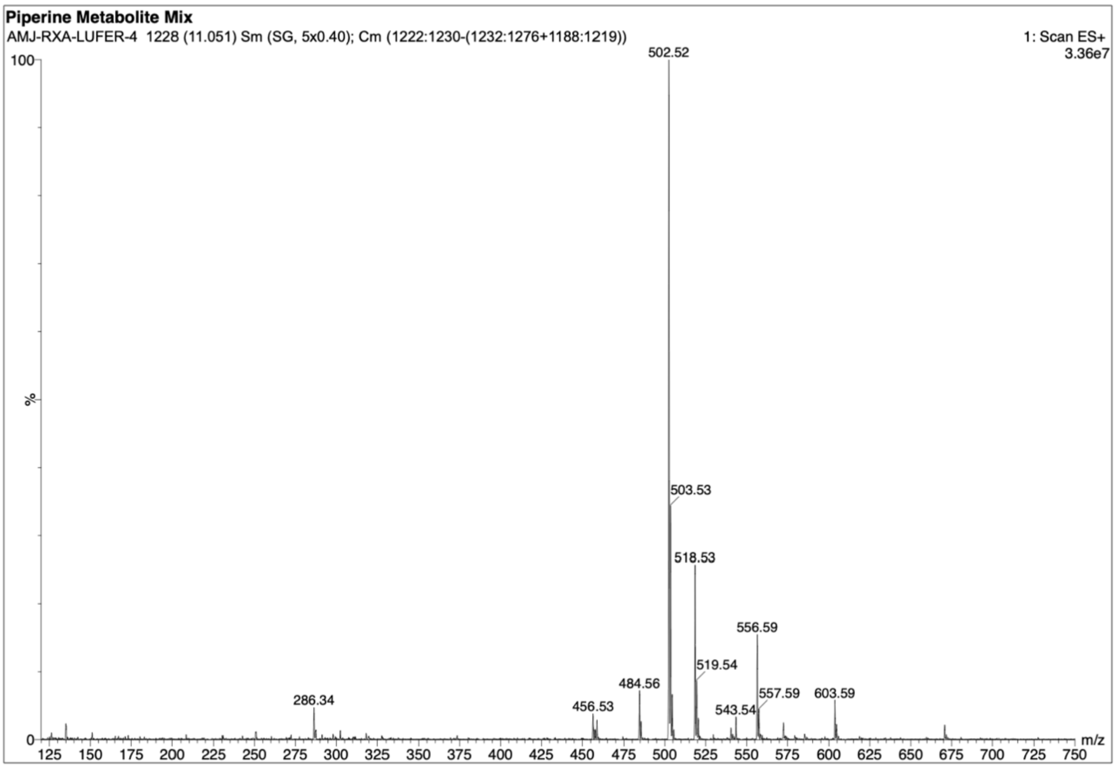

Metabolite 9

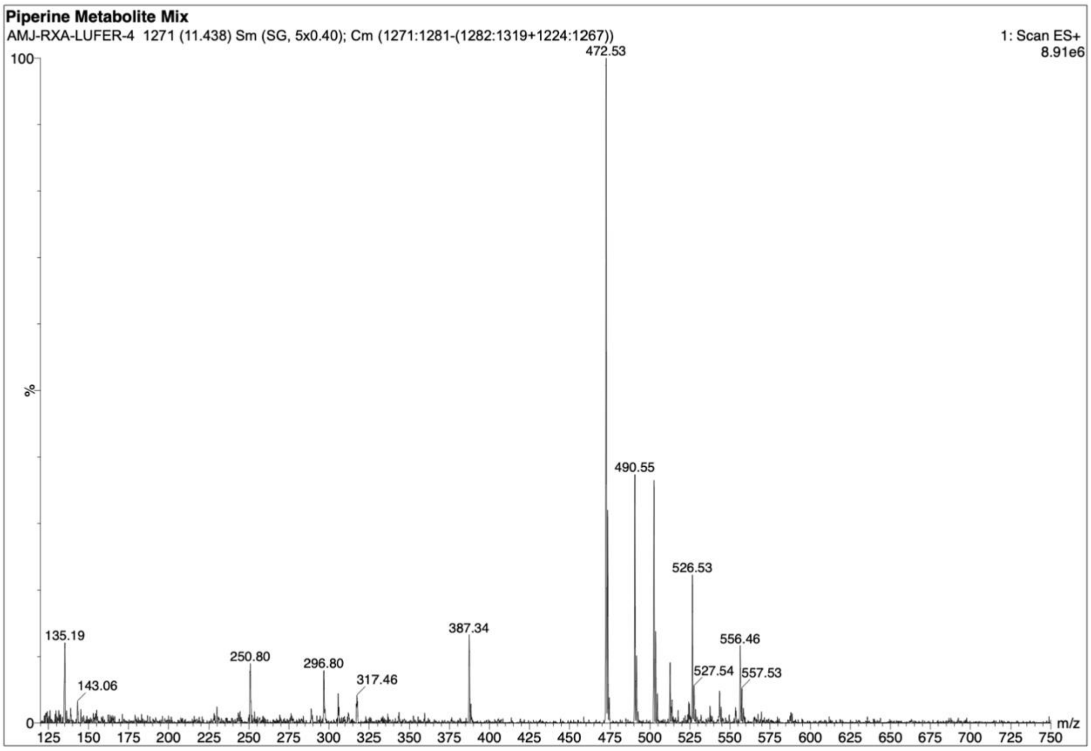

Metabolite 10

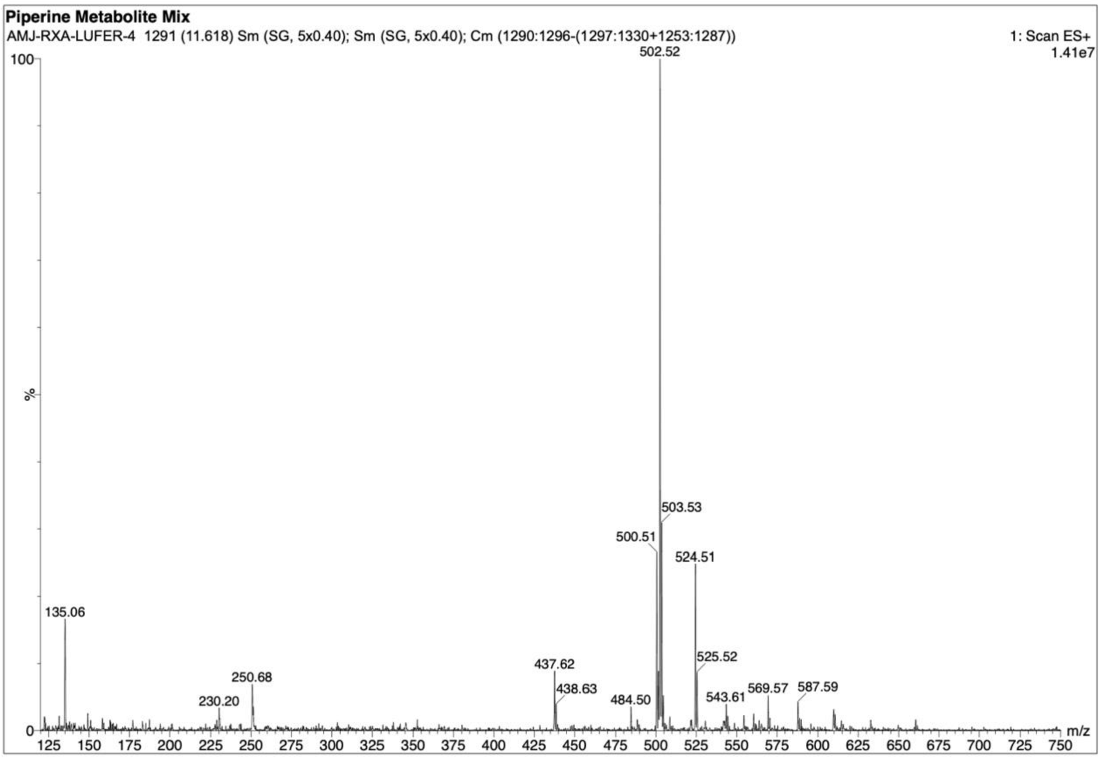

Metabolite 11

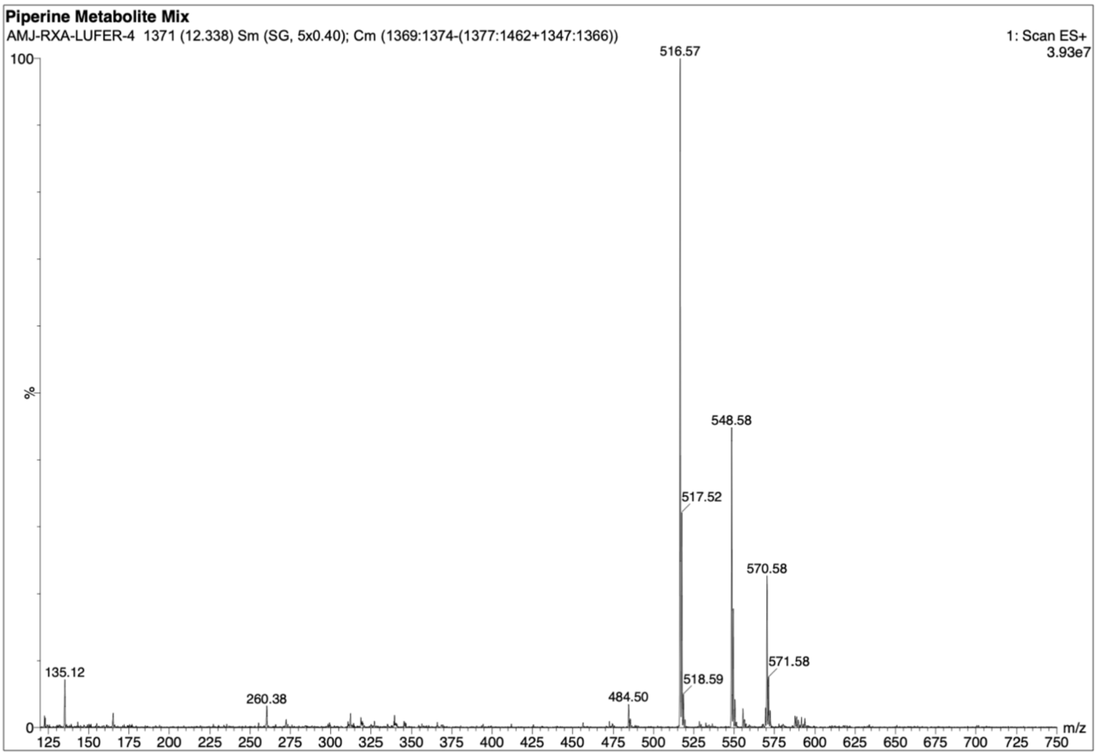

Metabolite 12

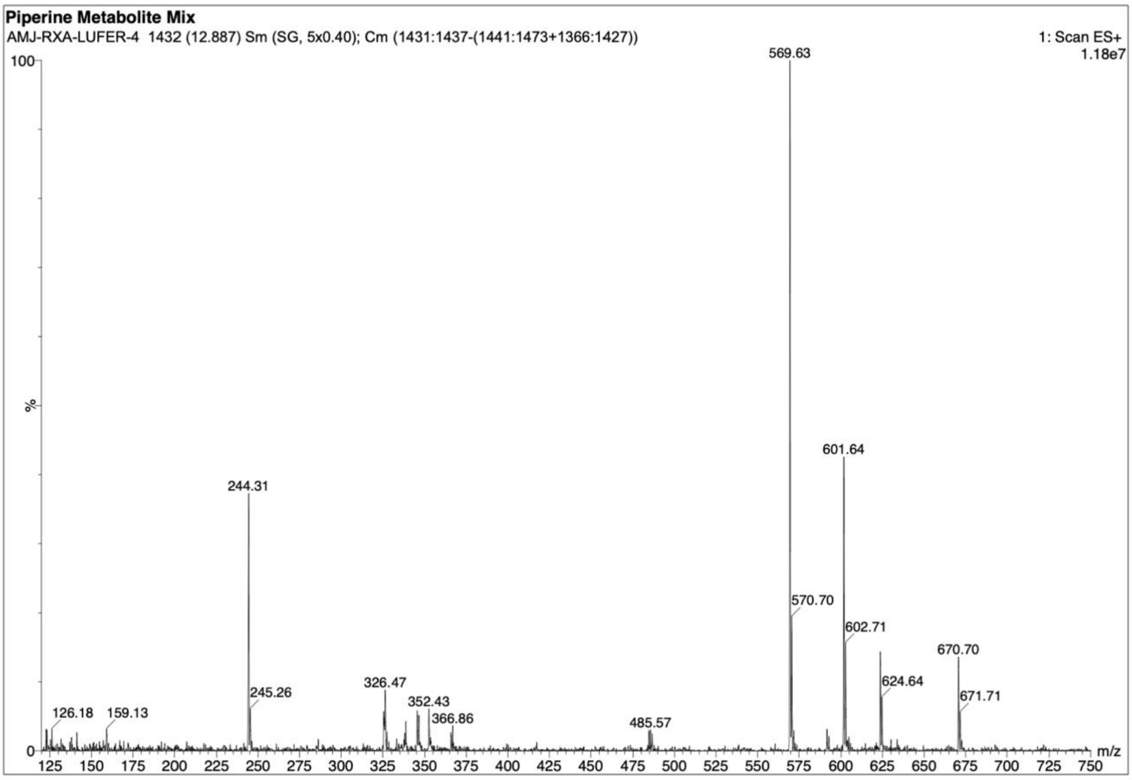

Metabolite 13

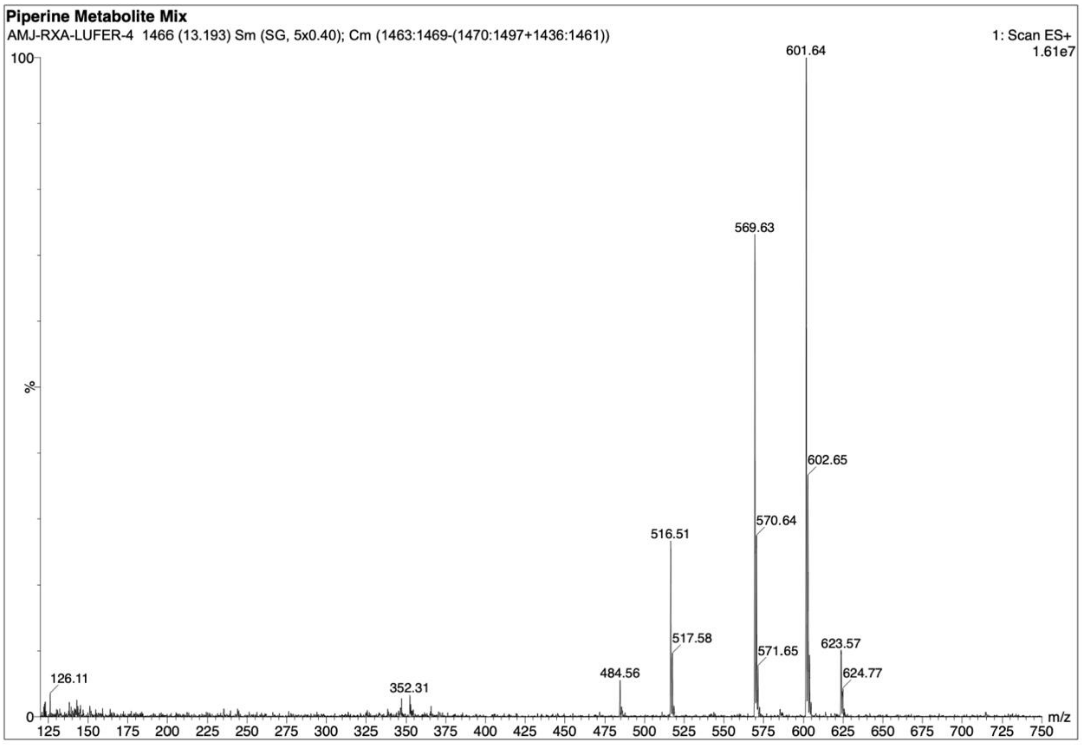

Metabolite 14

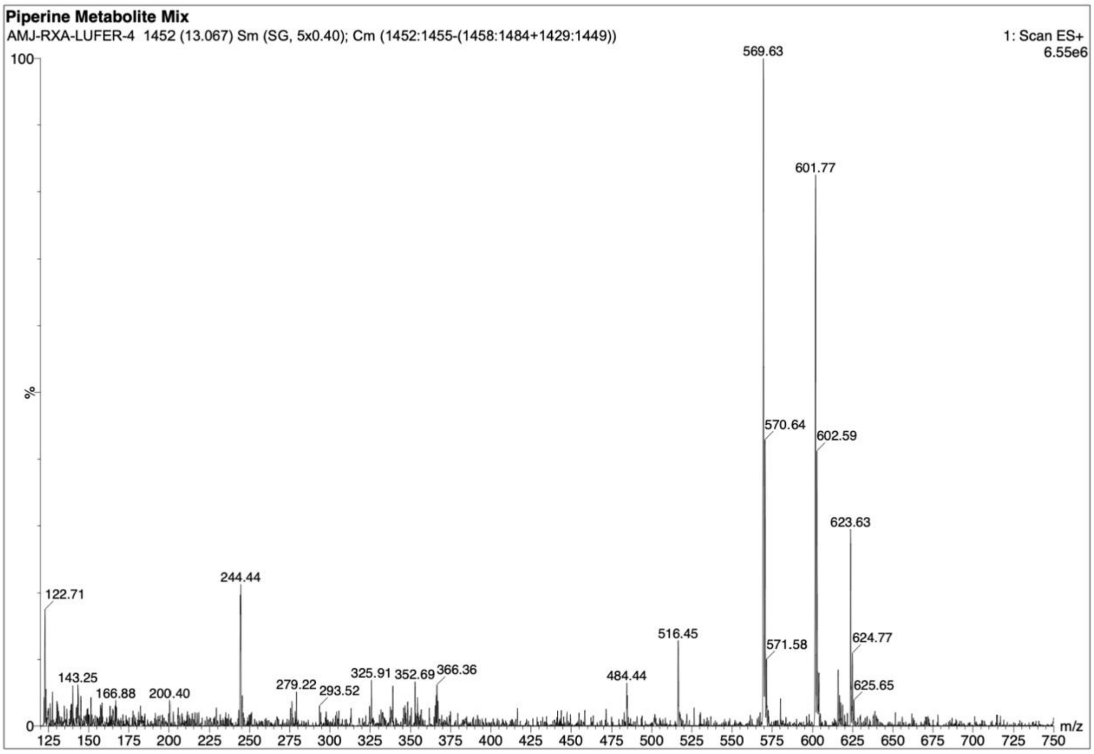

Piperine Standard analysis

1.	TLC of Piperine standard	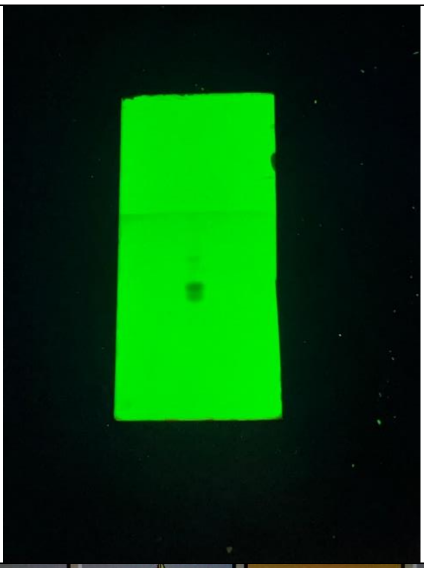
2.	Purification of the standard using flash column chromatography Biotage® Isolera™ Systems (SiO_2, eluent - Cyclohexane: EtOAc – 80 :20)	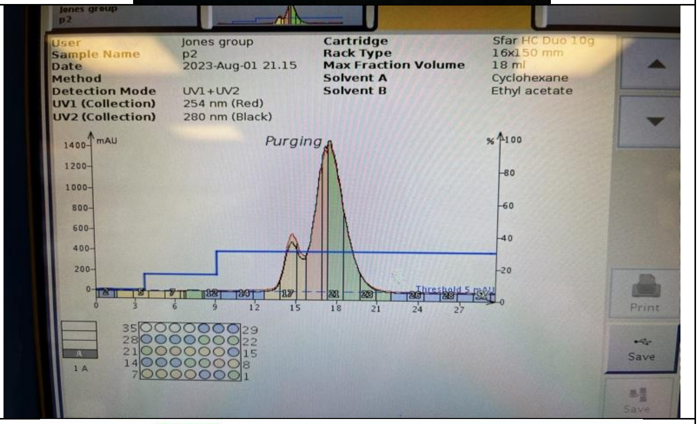
3.	*Results*	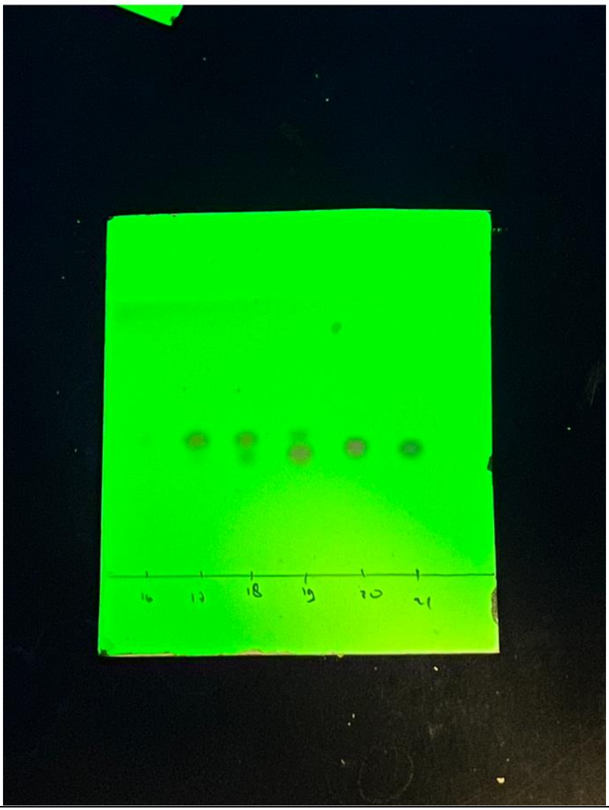

		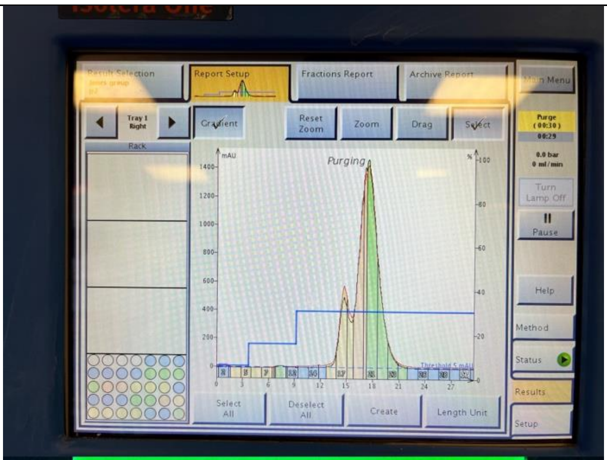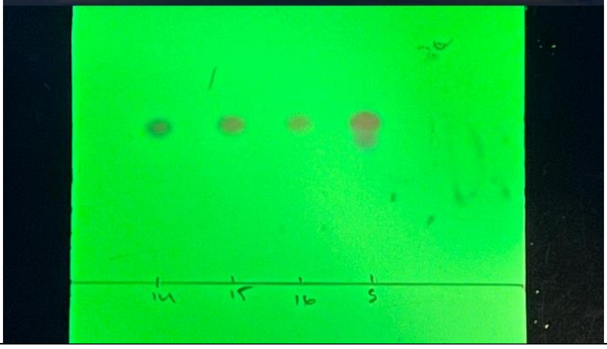
4.	*NMR of 1st spot*	*NMR showed a mixture of Piperine isomer (confirmed by LCMS)*
5.	*LCMS analysis*	*LCMS analysis showed the same molecular weight (piperine)*

NMR of 1st spot

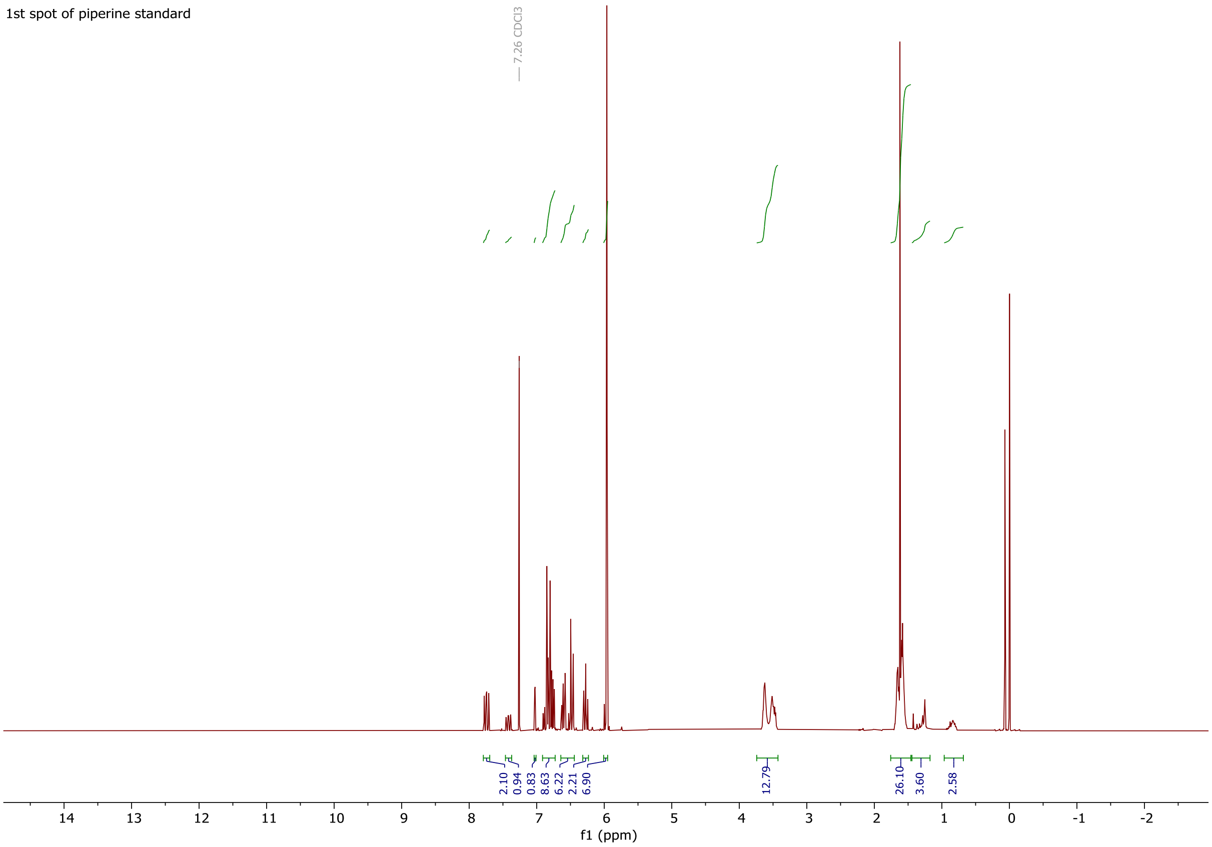

LCMS Analysis

1. Blank analysis

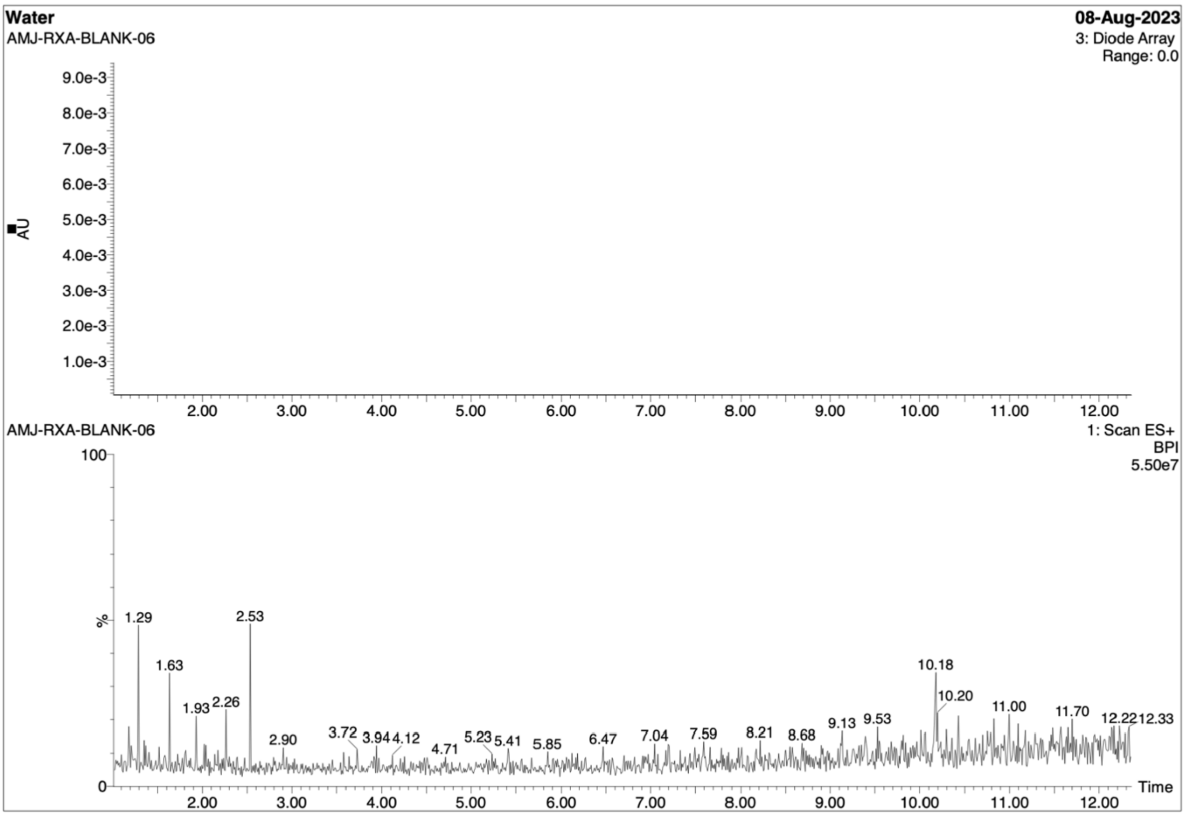

2. Piperine standard

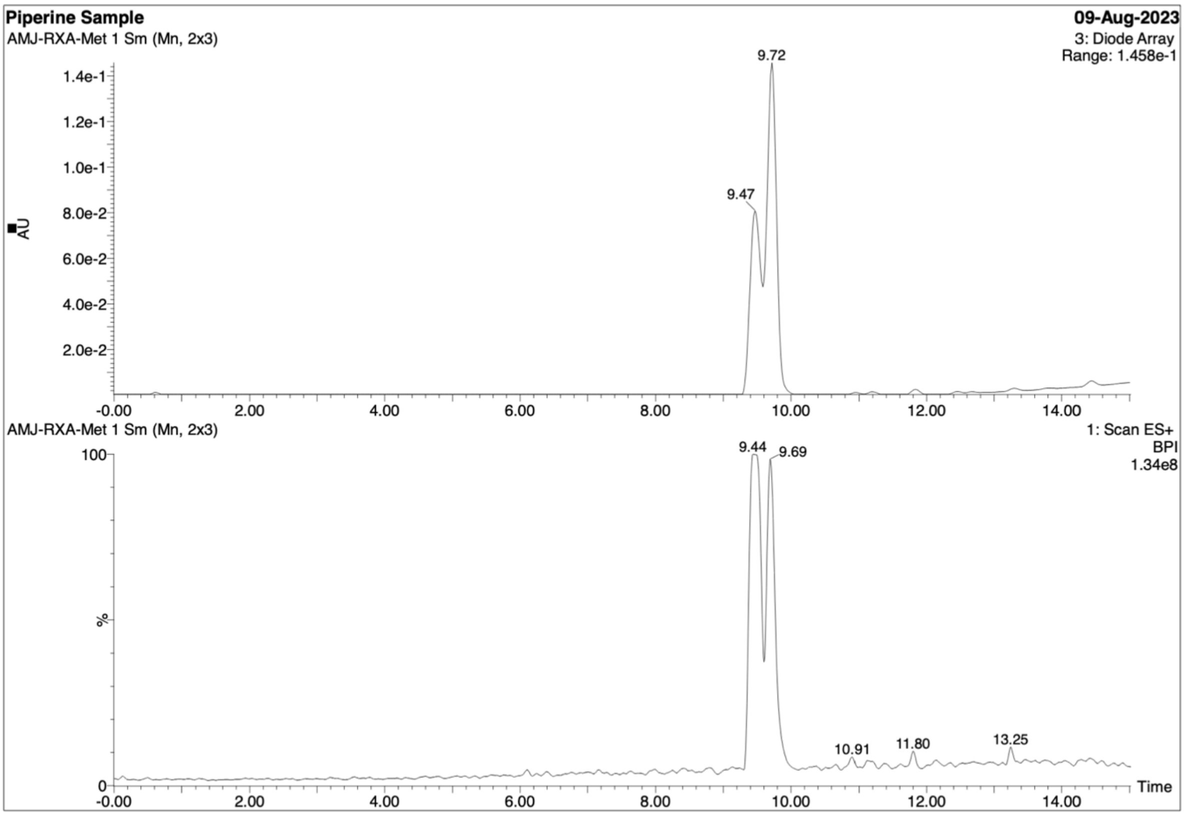

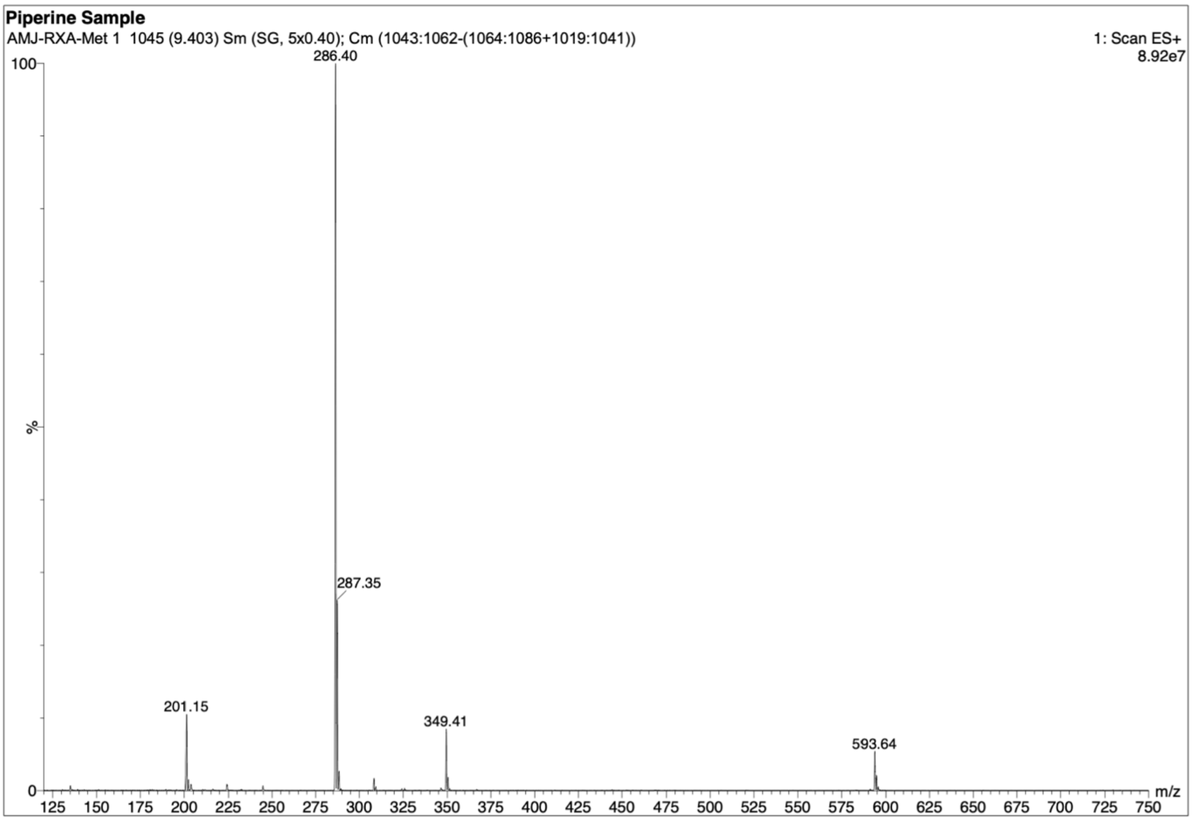

3. 1st spot

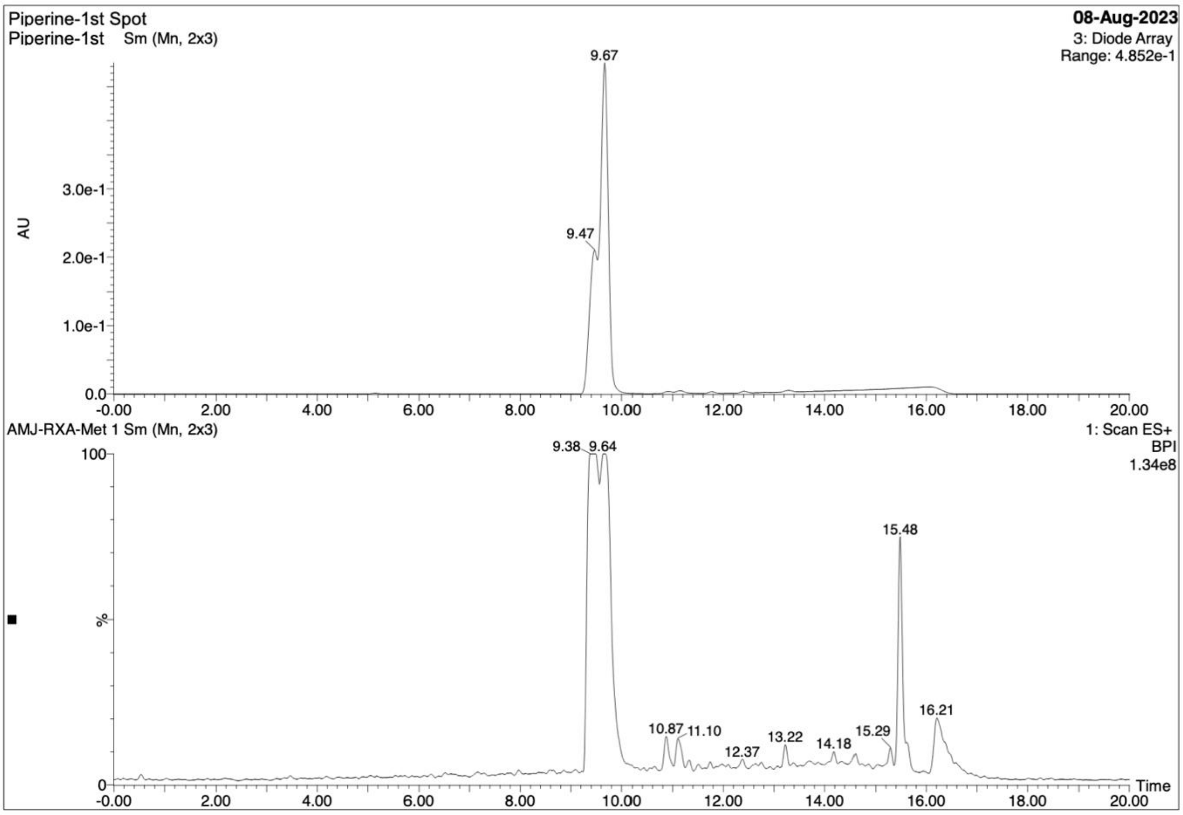
Piperine-1st Spot
Piperine-1st Sm (Mn, 2x3)
08-Aug-2023
3: Diode Array
Range: 4.852e-1
9.67
9.47
AU
3.0e-1
2.0e-1
1.0e-1
0.0
-0.00
2.00
4.00
6.00
8.00
10.00
12.00
14.00
16.00
18.00
20.00
AMJ-RXA-Met 1 Sm (Mn, 2x3)
1: Scan ES+
BPI
1.34e8
9.38
9.64
15.48
16.21
10.87
11.10
12.37
13.22
14.18
15.29
100
%
0
Time

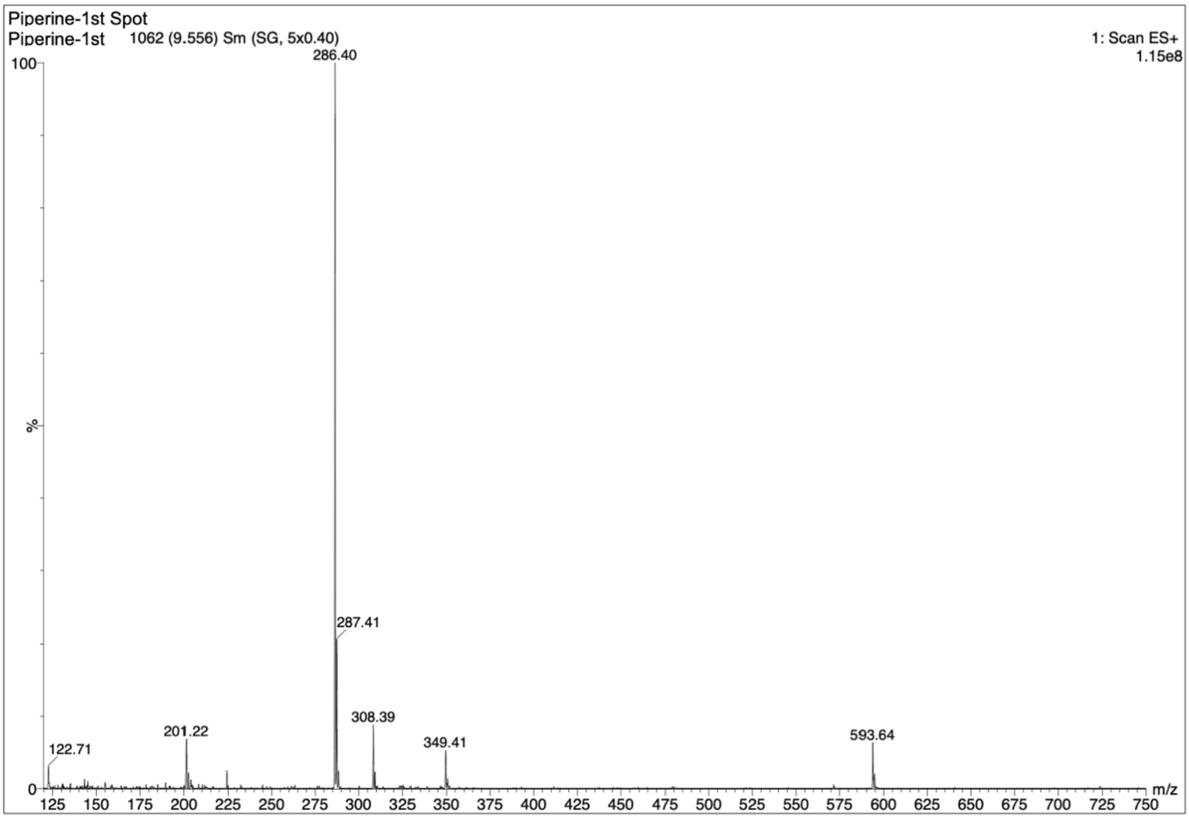
Piperine-1st Spot
Piperine-1st 1062 (9.556) Sm (SG, 5x0.40)
1: Scan ES+
1.15e8
286.40
100
%
0
287.41
308.39
349.41
201.22
122.71
593.64
m/z
125 150 175 200 225 250 275 300 325 350 375 400 425 450 475 500 525 550 575 600 625 650 675 700 725 750

Cyclic voltammetry studies of piperine

1.1.1. Piperine

Preliminary Analysis

Blank/control solution analysis
(0.1 M tetrabutylammonium hexafluorophosphate in 10 mL MeCN)

CV staircase setting:
Start potential: 0 Vreff
Upper Vertex Potential: 3.00 Vreff
Lower Vertex Potential: -0.10 Vreff Stop Potential: 0 Vreff
Number of Scans: 1
Scan Rate: 0.20 V/s
Step: 0.00244 V

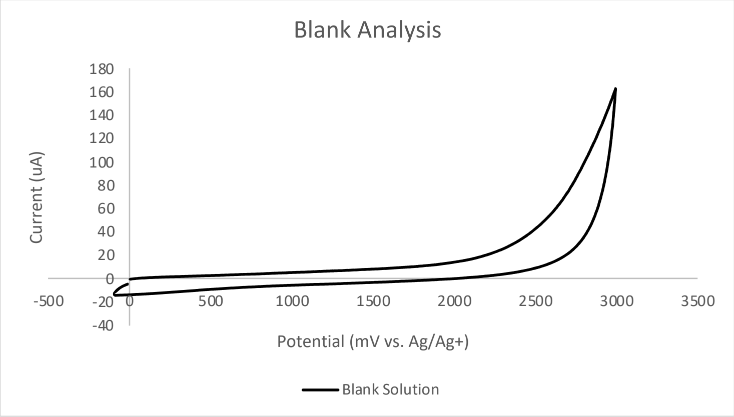

Reference solution
(1 mM ferrocene and 0.1 M tetrabutylammonium hexafluorophosphate in 10 mL MeCN)

CV staircase setting:
Start potential: 0 Vreff
Upper Vertex Potential: 0.90 Vreff
Lower Vertex Potential: -0.20 Vreff
Stop Potential: 0 Vreff
Number of Scans: 1
Scan Rate: 0.20 V/s
Step: 0.00244 V

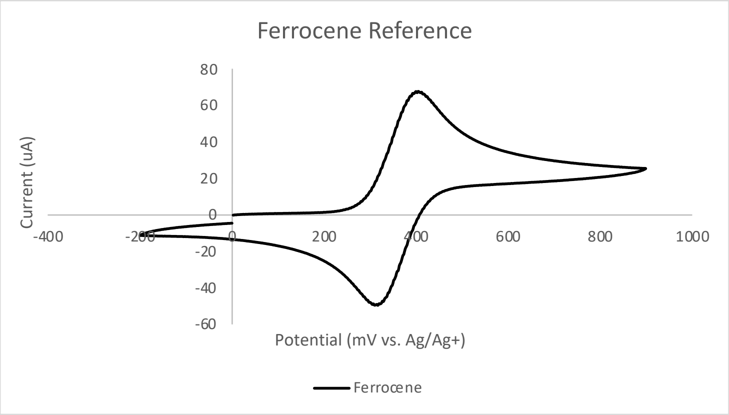

Ep 0.407 V; Ep_2 0.312 V; $E_{1/2}$ 0.36 V

Potential Range of Piperine

CV staircase setting:
Start potential: 0 Vreff
Upper Vertex Potential: 3.00 Vreff
Lower Vertex Potential: -0.10 Vreff
Stop Potential: 0 Vreff
Number of Scans: 1
Scan Rate: 0.20 V/s
Step: 0.00244 V

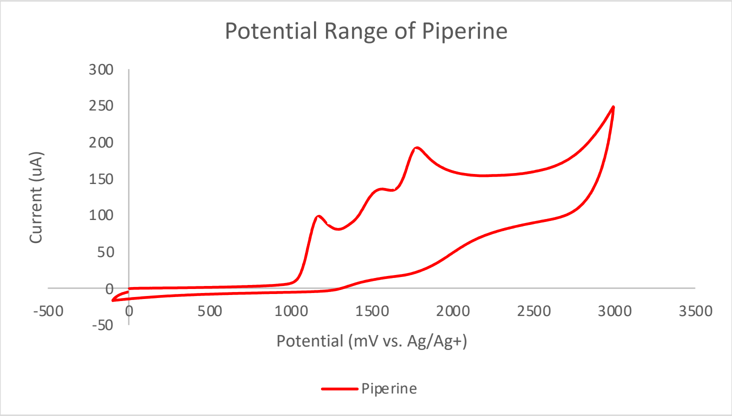

CV staircase setting:
Start potential: 0 Vreff
Upper Vertex Potential: 3.00 Vreff
Lower Vertex Potential: -0.10 Vreff
Stop Potential: 0 Vreff
Number of Scans: 3
Scan Rate: 0.20 V/s
Step: 0.00244 V

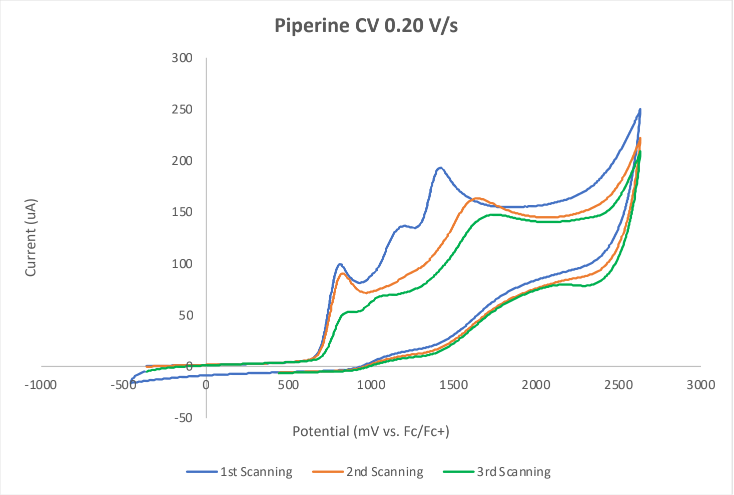

Cyclic Voltammetry Studies of Piperine

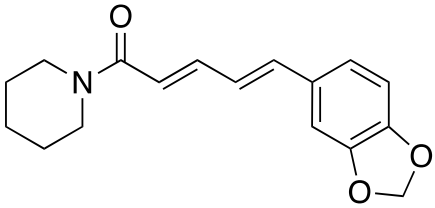

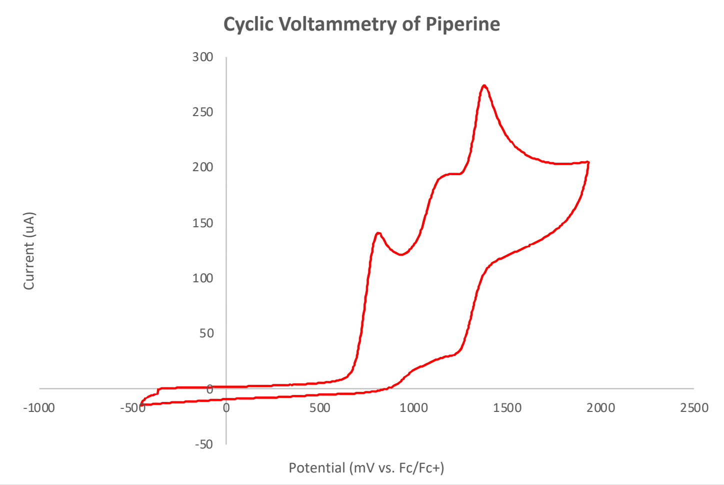

CV staircase setting:
Start potential: 0 Vreff
Upper Vertex Potential: 2.30 Vreff
Lower Vertex Potential: -0.10 Vreff
Stop Potential: 0 Vreff
Number of Scans: 1
Scan Rate: 0.20 V/s
Step: 0.00244 V

The variation in concentrations
CV staircase setting:
Start potential: 0 Vreff
Upper Vertex Potential: 2.30 Vreff
Lower Vertex Potential: -0.10 Vreff
Stop Potential: 0 Vreff
Number of Scans: 1
Scan Rate: 0.20 V/s
Step: 0.00244 V

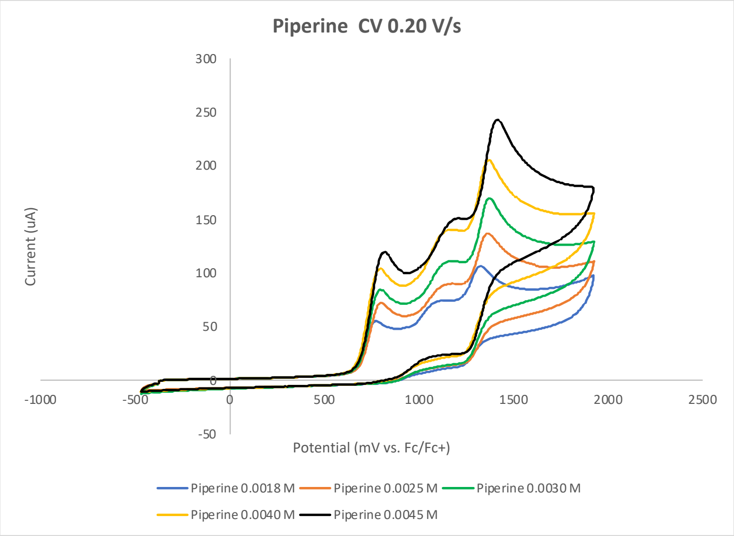

Piperine 0.0018 M: Ep 0.745 V; ip 58 μA.
Piperine 0.0025 M: Ep 0.766 V; ip 75 μA.
Piperine 0.0030 M: Ep 0.789 V; ip 84 μA.
Piperine 0.0040 M: Ep 0.779 V; ip 103 μA.
Piperine 0.0045 M: Ep 0.805 V; ip 118 μA.

The effect of scan rate
CV staircase setting:
Start potential: 0 Vreff
Upper Vertex Potential: 2.30 Vreff
Lower Vertex Potential: -0.10 Vreff
Stop Potential: 0 Vreff
Number of Scans: 1
Scan Rate: 0.05-0.30 V/s
Step: 0.00244 V

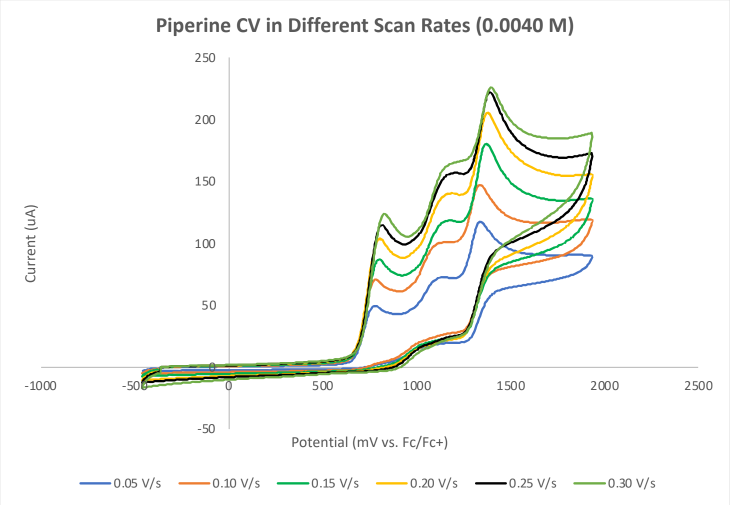

SR 0.05 V/s: Ep 0.740 V; ip 50 μA.

SR 0.10 V/s: Ep 0.759 V; ip 72 μA.

SR 0.15 V/s: Ep 0.771 V; ip 87 μA.

SR 0.20 V/s: Ep 0.788 V; ip 105 μA.

SR 0.25 V/s: Ep 0.801 V; ip 115 μA.

SR 0.30 V/s: Ep 0.818 V; ip 123 μA.

CV Quadruple Scanning
CV staircase setting:
Start potential: 0 Vreff
Upper Vertex Potential: 2.30 Vreff
Lower Vertex Potential: -0.10 Vreff
Stop Potential: 0 Vreff
Number of Scans: 4
Scan Rate: 0.25 V/s
Step: 0.00244 V

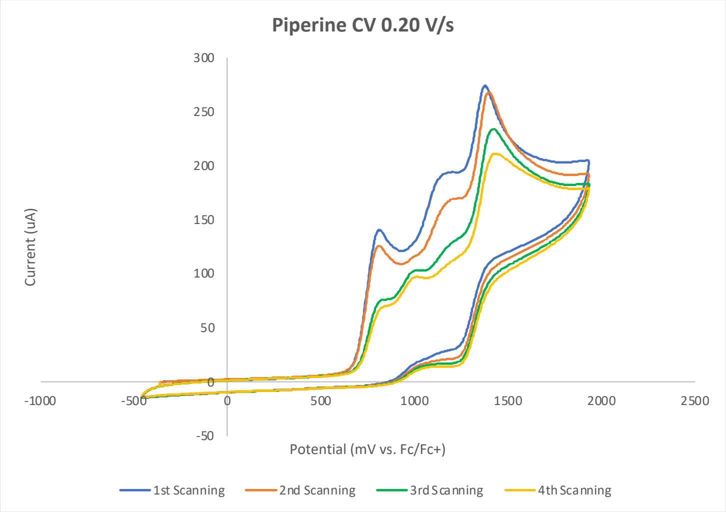

1st Scanning
Ep 0.828 V; ip 139 μA
2nd Scanning
Ep 0.801 V; ip 125 μA
3rd Scanning
Ep 0.840 V; ip 76 μA
4th Scanning
Ep 0.850 V; ip 70 μA

The electron involved in reaction NA (the electron involved in reaction can be calculated if the value of Ep and Ep_2 are available).

Log of Peak Potential (Ep) Log of Scan Rate (mV/s)

y = 0.0539x + 2.7743
R^2 = 0.9503

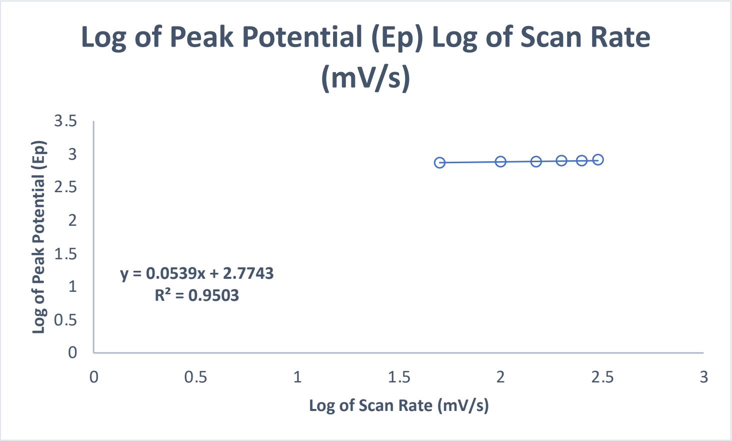

Log of Peak Current (ip) Log of Scan Rate (mV/s)

y = 0.5111x + 0.8329
R^2 = 0.9503

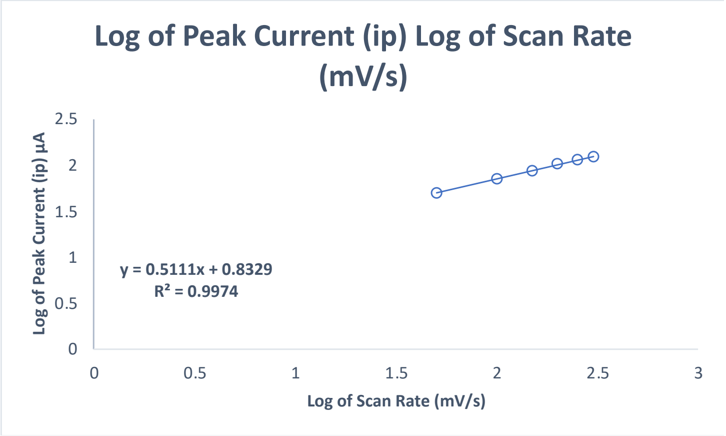

References

[S1] Zazeri, G.; Povinelli, A.P.R.; Lima, M.d.F.; Cornélio, M.L. Experimental Approaches and Computational Modeling of Rat Serum Albumin and Its Interaction with Piperine. *Int. J. Mol. Sci.* **2019**, *20*, 2856. https://doi.org/10.3390/ijms20122856
